# Distribution and voltage dependence of ion channels shape single-neuron computations

**DOI:** 10.1073/pnas.2610600123

**Published:** 2026-09-29

**Authors:** Renée M. Vieira, Michael Deistler, Clémentine Guillemet, Tabea Schilling, Jakob H. Macke, Alexander Borst, Lukas N. Groschner

**Affiliations:** ^a^ Max Planck Institute for Biological Intelligence, Martinsried 82152, Germany; ^b^ Machine Learning in Science, University of Tübingen, Tübingen 72076, Germany; ^c^ Tübingen AI Center, Tübingen 72076, Germany; ^d^ Department Empirical Inference, Max Planck Institute for Intelligent Systems, Tübingen 72076, Germany; ^e^ Gottfried Schatz Research Center, Molecular Biology and Biochemistry, Medical University of Graz, Graz 8010, Austria

**Keywords:** *Drosophila*, neuroscience, ion channel, endogenous tagging, computational model

## Abstract

Voltage-gated ion channels do not distribute evenly in the plasma membrane of a neuron; they localize to distinct domains where they govern the excitability of the cell and the computations it performs. Informed by electron microscopic reconstructions, models of morphologically realistic neurons offer the opportunity to study these computations, but they lack constraints on the subcellular localizations and functional properties of voltage-gated channels. Here, we address this problem in *Drosophila melanogaster* by developing tools to tag and visualize endogenous ion channels in genetically designated populations of cells and in single neurons. Focusing on a suite of highly expressed ion channels in visual motion-sensitive neurons, we report their subcellular localizations and their steady-state activation and inactivation curves. Integrating these structural and functional data into a biophysically constrained model reproduces the directional tuning of motion-sensitive T5 neurons and reveals the function of voltage-gated sodium channels in nonspiking neurons.

## Significance

The computations performed by a single neuron depend on the function and the subcellular localization of voltage-gated ion channels. We develop in *Drosophila* a set of tools to map endogenous channels in genetically defined cells and characterize their currents in patch-clamp experiments. Constrained by these data, we build a biophysically realistic model of the motion vision circuit. Our simulations reveal a persistent “window” current through axonal sodium channels. This current generates a robust nonlinearity that sharpens the directional tuning of motion-sensitive neurons, demonstrating how nonspiking neurons leverage voltage-gated channels to perform complex computations.

Connectomics has revolutionized neuroscience by delivering synaptic wiring diagrams of entire nervous systems alongside intricate three-dimensional reconstructions of neuronal morphologies (1–6). More often than not, the function of a neuron is inextricably linked to its shape (7); yet, connectome-inspired computational models are based predominantly on synaptic connectivity matrices and disregard neuronal morphology (8, 9). They usually sacrifice microanatomical and biophysical detail in favor of computational efficiency and practicability. This compromise leaves us blind to computational feats that emerge from cellular structure and the strategic positioning of ion channels.

Connectivity alone is not enough to capture neural computations. Across phyla, critical operations—such as temporal filtering (10–12), multiplicative processing (13), and synaptic integration (14–17)—rely on voltage-gated ion channels that sit on neurites of varying shapes. Constructing active multicompartment models that capture these shapes and gating functions is challenging because they involve large numbers of unconstrained parameters. Recently, accelerated by the computational efficiency of graphics processing units, multicompartmental multi-neuron models are gaining momentum (18). Such biophysically realistic simulations raise hopes of revealing relationships between neuronal form and function (19); especially in *Drosophila*, where connectomes are nearly complete and plentiful (1–6). But to live up to this promise, realistic compartmental models require at least three additional pieces of information: the identities of the voltage-gated channels present, their gating characteristics, and their exact subcellular localizations.

In this study, we search for these missing constraints. We provide a genetic toolkit for visualizing the subcellular distribution of endogenous ion channels and a reference compendium of their functional properties. To showcase the capabilities of these methods, we use them to characterize the ion channel landscapes of direction-selective T4 and T5 neurons (20). By combining structural and functional data, we construct a biophysically detailed model which identifies a mechanism that sharpens the directional tuning of these cells.

## Results

### Subcellular Channel Distribution Dictates Spike Patterns.

**Fig. 1.**
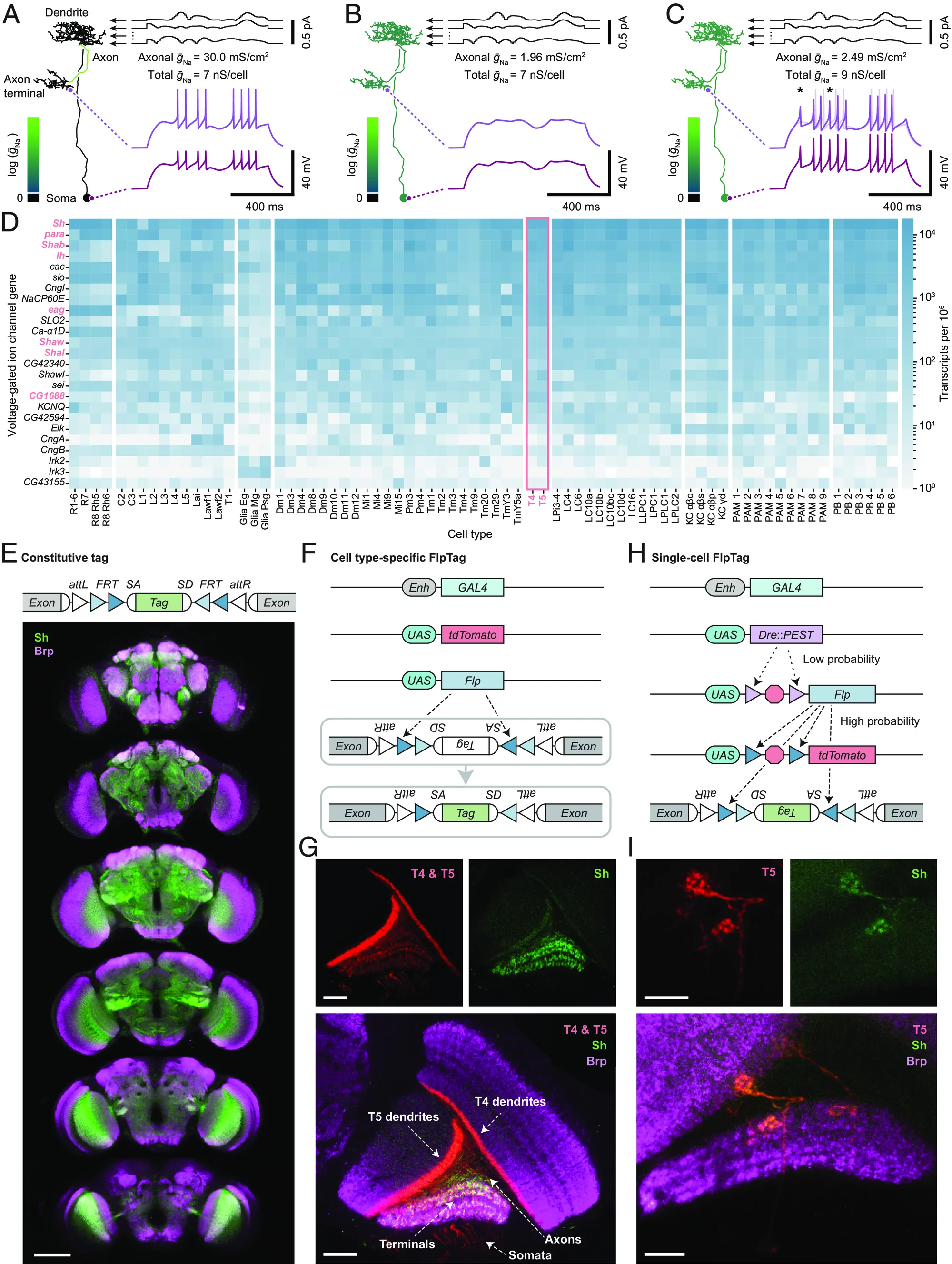
Three ways of tagging endogenous ion channels. (*A*) Hodgkin–Huxley-type compartmental model of a T5 neuron with sodium channels spatially confined to the axon. Fluctuating depolarizing currents injected into 32 different dendritic locations (*Top*) evoked distinct spike patterns that propagated to the axon terminal (purple) and the soma (magenta). Neurite color corresponds to local sodium conductance density (*ḡ*_Na_). (*B*) Same as (*A*) for a homogenous distribution of sodium channels. Dendritic current injections did not elicit spikes despite identical total *ḡ*_Na_. (*C*) The conductance of uniformly distributed sodium channels was tuned to reproduce the activity pattern of (*A*). The fitted model failed to capture spike times accurately and generated extra spikes (asterisks). The shaded line shows original membrane potential from (*A*). (*D*) Ion channel gene transcripts in *Drosophila* neurons and glia sorted by increasing abundance. Data from Davis et al. (22). Marked in red are tagged channels on the ordinate and T4 and T5 neurons on the abscissa. (*E*) *Top*: Constitutive tags, flanked by splice acceptor (SA) and splice donor sites (SD), integrate as artificial exons and label the endogenous protein unconditionally. Light and dark blue triangles indicate *FRT* and *FRT14* flippase recognition target sites, respectively. *Bottom*: Endogenous Shaker potassium channels (Sh, green) and presynaptic Bruchpilot (Brp, magenta) in frontal confocal cross sections of a *Drosophila* brain. (Scale bar, 50 µm.) (*F*) Conditional FlpTag constructs harbor the tag-bearing cassette in the antisense orientation. A GAL4 driver (*Enh–GAL4*) targets tdTomato and the recombinase flippase (Flp) to a genetically defined population of cells, where it inverts the *FlpTag* (gray arrow). (*G*) Horizontal confocal cross-section of the *Drosophila* optic lobe. T4 and T5 neurons are shown in red, endogenously tagged Sh in green, and Brp in magenta. *Top*: individual channels; *Bottom*: overlay. (Scale bars, 20 µm.) (*H*) Single-cell FlpTag allows for the labeling of endogenous proteins in a sparse selection of cells within a population. It uses an additional recombinase with a short half-life (*Dre::PEST)* to stochastically drive expression of *tdTomato* and *Flp*. (*I*) Horizontal confocal cross-section through the optic lobe. Single T5 neurons are shown in red, endogenously tagged Sh in green, and Brp in magenta. *Top*: individual channels; *Bottom*: overlay. (Scale bars, 10 µm.)

To illustrate how the spatial distribution of ion channels influences neural activity, we first built a Hodgkin–Huxley-type multicompartmental model (18) using the anatomical skeleton of a T5 neuron from the visual system of the fly (21) (Fig. 1 *A*–*C*). All compartments of the model neuron contained a constant leak conductance and a delayed-rectifier potassium channel. Voltage-gated sodium channels, however, were placed at varying locations: in the first scenario, they were confined to the axon, where we increased their conductance until we observed a spiking response to dendritic input currents (Fig. 1*A*). In the second scenario, we used the same sodium conductance that generated action potentials when inserted in the axon, but distributed it evenly across the entire neuron. After the redistribution, the neuron remained silent in response to the dendritic stimulus, even though the total sodium conductance was conserved (Fig. 1*B*). The attempt to recover the original spike train by fitting the magnitude of the uniformly distributed sodium conductance failed owing to the persistent emergence of aberrant spikes and imprecise spike timing (Fig. 1*C*). An analogous model of a T4 neuron painted a similar picture (*SI Appendix*, Fig. S1). With the altered, homogenous channel distribution, it proved impossible to reproduce the original spike pattern. This ingenuous computational thought experiment demonstrates that it is not sufficient to know the total abundances of ion channels; their subcellular localizations are crucial determinants of a neuron’s output.

### Voltage-Gated Ion Channels of the *Drosophila* Brain.

Constraining ion channel localizations requires methods to visualize native channels in genetically defined neurons. The genome of *Drosophila melanogaster* contains approximately 25 genes encoding voltage-gated ion channels; a strikingly sparse complement compared to mammals (23). In the fly, functional diversity comes from extensive alternative splicing. A conditional tag inserted strategically into a portion of an ion channel gene common to all isoforms, therefore, offers an effective approach for visualizing most—if not all—of its splice variants.

We curated a published transcriptomic dataset (22) to profile the expression of channel genes across 74 genetically designated cell types in the adult *Drosophila* brain. To maintain a focus on primary excitability, our inventory was restricted to transcripts encoding the pore-forming α subunits of canonical voltage-gated sodium, potassium, and calcium channels and some related families that exhibit voltage-dependent rectification or gating, like cyclic nucleotide-gated channels, calcium-activated potassium channels, and channels with voltage-dependent rectification. Our selection excluded auxiliary β subunits, voltage-independent leak channels, antiporters, pumps, intracellular channels, gap junction genes, ligand-gated channels, and ionotropic neurotransmitter receptors, whose subcellular localizations have been the focus of recent investigations (24–26).

Among the 25 transcripts of interest, potassium channels—confirmed or putative—constituted a 64% majority. Channel transcripts were generally more abundant in neurons than in glia (Fig. 1*D*). This overview formed a blueprint for prioritizing which genes to target in subsequent experiments.

### Genetic Tools for the Constitutive and Conditional Tagging of Ion Channels.

Guided by this inventory, we used ΦC31 recombinase-mediated cassette exchange (27) to insert epitope and fluorescent tags into intronic regions of eight highly expressed channel genes (Fig. 1*D* and *SI Appendix,* Table S1). *Minos*-mediated integration cassettes (MiMIC) (28) and CRISPR-mediated integration cassettes (CRIMIC) (29) served as vehicles to introduce one of three genetically encoded tags: three small simian virus 5-derived epitopes (V5), enhanced green fluorescent protein (EGFP), or a high-sensitivity “spaghetti monster” tag (30) decorated with twelve V5 epitopes (smV5). While the choice of tag influenced the sensitivity of detection, it did not alter the subcellular trafficking or localization of the target protein (*SI Appendix*, Fig. S2*A*).

At its core, our endogenous tagging approach relies on a conditional genetic switch. The integrated cassettes carry a splice donor and acceptor sequence on opposite sides, flanked by a duet of inverted *FRT* and *FRT14* flippase recognition target sites (Fig. 1*E* and *SI Appendix*, Fig. S2*B*). The cassettes integrate in either of two orientations: 1) The constitutive orientation, which leads to the seamless translation of the tag as an artificial exon (Fig. 1*E*) and offers an overview of endogenous channel expression and localization across cell types and organs (e.g., the entire brain, Fig. 1*E*); and 2) the conditional *FlpTag* orientation, in which the intronic tag is removed by default during RNA splicing. Under these conditions, translation of the *FlpTag* depends on the inversion of the cassette by the recombinase flippase (Flp). Once introduced via the *GAL4/UAS* system, Flp inverts the tag and excises the inverted FRT site locking the tag in its translatable, active orientation (28, 31) (*SI Appendix*, Fig. S2*B*). This leads to irreversible labeling of the endogenous ion channel in a genetically defined population of cells (Fig. 1*F*).

Putting this approach to the test, we used *R42F06-GAL4* to target *Flp* expression to T4 and T5 neurons in the *Drosophila* visual system. These motion-sensitive cells have stereotyped morphologies, and their ion channel transcriptomes reflect the brain-wide average (22) (Fig. 1*D*). We started by tagging the *Shaker* (*Sh*) gene, which encodes the prototypical A-type potassium channel (32) and represents the most abundant channel transcript in these cells (Fig. 1*D*). Endogenous Sh protein was highly enriched in the axon terminals and distal axons, present at lower levels in the dendrites, and entirely absent from the somata and primary neurites (Fig. 1*G*).

Because neurites are densely interwoven, tagging a channel in a population of cells does not always provide a clear picture of its subcellular distribution. To single out individual neurons among genetically defined populations, we devised two complementary strategies involving cascades of recombinases. The first method uses the moderately efficient Dre recombinase (33) destabilized by a *PEST* sequence (34) to excise a stop cassette with low probability. This stochastic event gives way to the GAL4-driven expression of *Flp*—and thus the activation of the *FlpTag*—only in a sparse fraction of cells within a large population, like T5 neurons (Fig. 1 *H* and *I*). To achieve single-cell resolution also in smaller populations, where stochastic methods are notoriously inefficient, we developed a second system that employs a heat shock promoter to induce the expression of KD recombinase (34). This allowed us to fine-tune the time point and the density of single-cell tagging by changes in temperature (*SI Appendix*, Fig. S2*C*), as demonstrated by the labeling of endogenous GluClα in the dendrites of a single T4 neuron (*SI Appendix*, Fig. S2*D*). Single-cell labeling tools, however, yielded acceptable signal-to noise ratios only when used to tag the products of highly expressed genes like *Sh*.

### Brain-Wide Mapping Reveals Diverse and Layered Ion Channel Landscapes.

**Fig. 2.**
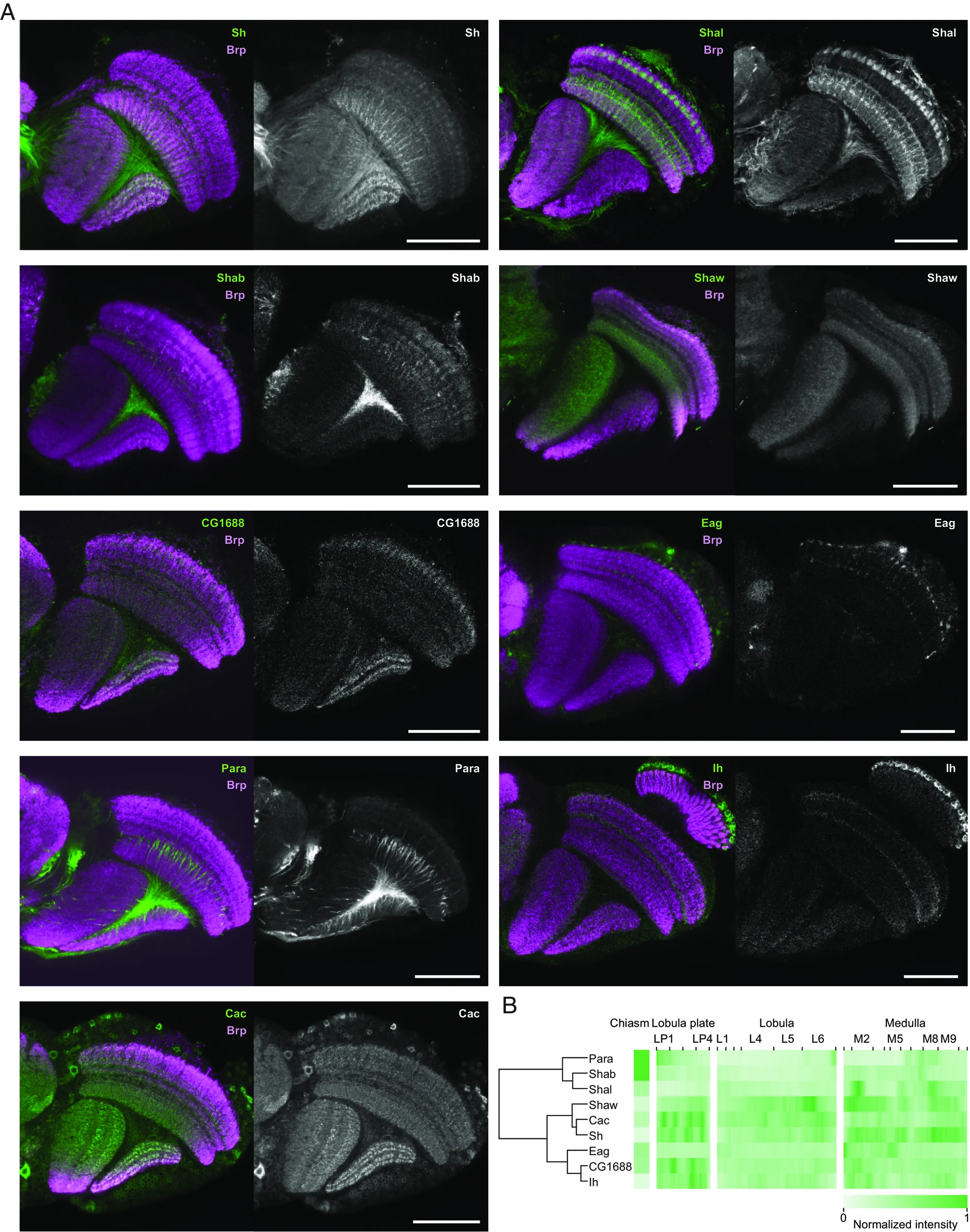
Differential localization of voltage-gated ion channels in the optic lobe. (*A*) Confocal cross sections of optic lobes with differential distributions of constitutively tagged ion channels (green) across neuropils (Brp, magenta). The localization of Cacophony (Cac) was visualized using an overexpression construct (36). (Scale bars, 50 µm.) (*B*) Hierarchical clustering of ion channel distributions based on normalized average green fluorescence intensity profiles (*n* = 5) along the longitudinal axes of the respective neuropils.

The *Drosophila* brain, and the exquisitely well-defined anatomy of the optic lobe (5, 6, 35) in particular, lent itself to a first assessment of channel distribution. We took confocal cross sections of brains and optic lobes of flies with eight different constitutively tagged voltage-gated channels, including the most abundant potassium, sodium, and nonselective cation channels (Fig. 2*A* and *SI Appendix*, Fig. S3). Because these transcripts are heavily biased toward neurons over glia (22) (Fig. 1*D*), the resulting immunofluorescence represents a predominantly neuronal map.

While eight endogenously tagged targets yielded robust signals, endogenous tagging of the voltage-gated calcium channel Cacophony (Cac) (37, 38) failed to produce detectable fluorescence despite multiple attempts. To visualize Cac, we resorted to a pan-neuronal driver to express a tagged construct (36), which mapped apart from synapses also onto somata (*SI Appendix*, Fig. S3). This distribution likely reflects a common artifact of overexpression (17, 24) rather than native trafficking and underscores the importance of tagging endogenous gene products in their native regulatory environments.

The endogenous maps revealed diverse, layer-specific patterns ranging from broad overlap to mutual exclusivity (Fig. 2 and *SI Appendix*, Fig. S3). In the optic lobe, Sh occupied the inner medulla, the axon tracts spanning the inner chiasm, and all four layers of the lobula plate, while sparing somata and the primary neurites that emerge from them. Conversely, its closest relative, Shaker cognate l (Shal) (39), was present in soma fibers and somata, in all layers of the inner, and layer M2 of the outer medulla as well as the inner chiasm. The primary voltage-gated sodium channel Paralytic (Para) (40, 41) and the delayed rectifying potassium channel Shaker cognate b (Shab) (39) were enriched in the chiasm and medullary layers M6 to M8 (Fig. 2*B*).

Hierarchical clustering of these global patterns also placed fast inactivating sodium and delayed rectifier potassium channels—the core ingredients for excitability (42)—in the same category. This physical proximity hints at a common biophysical mandate for Para and Shab, possibly in the generation of action potentials. Judging whether two channels actually cooperate to govern cellular excitability, however, requires moving past global distributions toward cell type–specific tagging to determine whether they reside in the membrane of the same cell.

### Localization and Function of Voltage-Gated Potassium Channels in T4 and T5 Neurons.

Many of the cells that criss-cross the chiasm are T4 and T5 neurons (43). Based on whole-cell recordings, they are thought to rely upon graded potentials rather than spike trains to transmit information (44–46). However, since whole-cell recordings can only be obtained from the (tiny) somata, and Para and Shab channels localize to the chiasm (Fig. 2), this leaves open the possibility that T4 and T5 cells, in fact, transmit spikes at their axon terminals. The inability to detect them at the soma might stem merely from voltage attenuation across their morphologies.

**Fig. 3.**
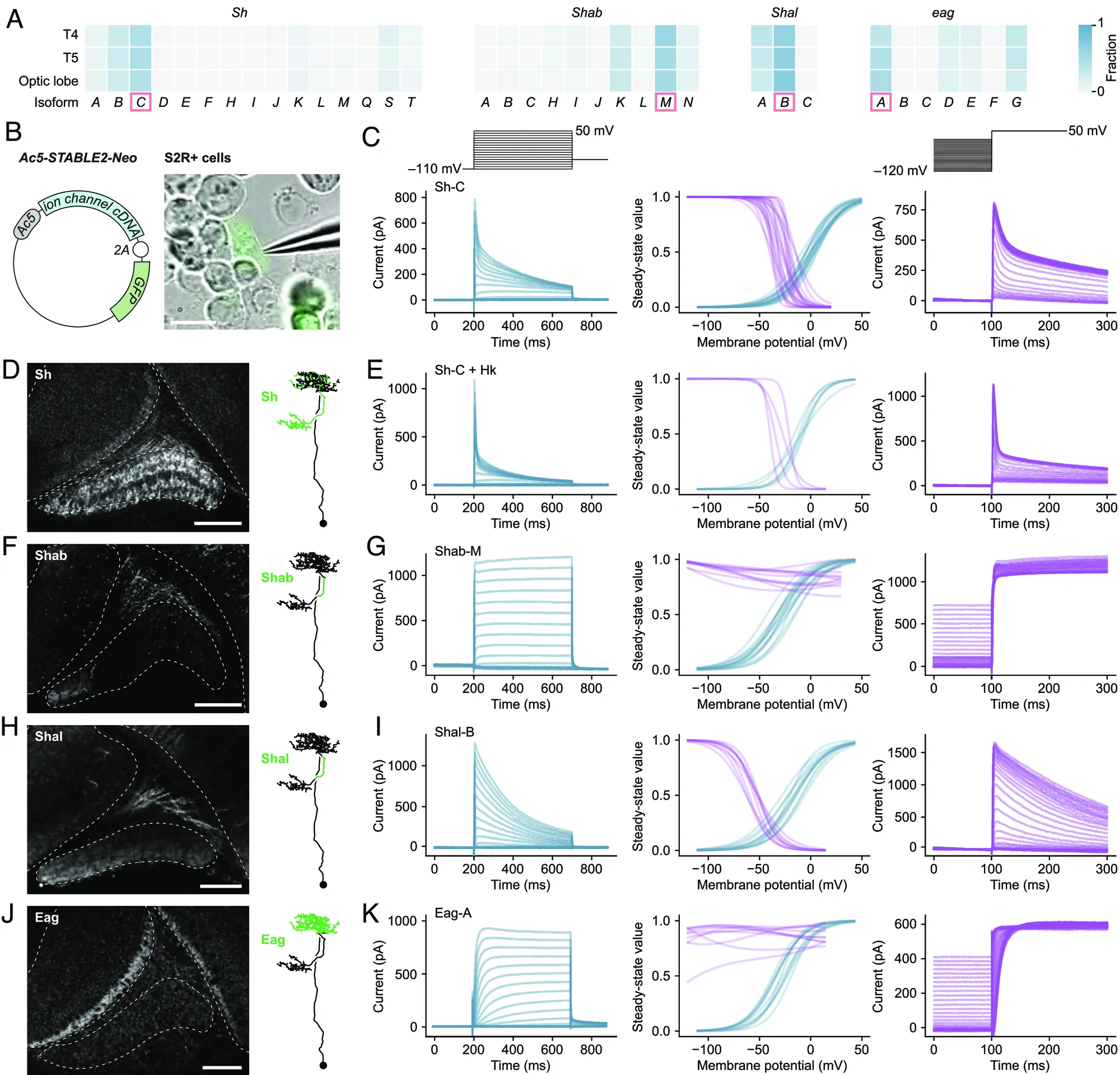
Potassium channels of T4 and T5 neurons. (*A*) Relative expression of potassium channel splice variants in T4 and T5 neurons and the optic lobe. Data from Davis et al. (22). Cloned isoforms are marked in red. (*B*) Expression of ion channel constructs (*Left*) was driven in S2R+ cells (*Right*) by the Actin-5C promoter (Ac5) and coupled to the expression of green fluorescent protein (GFP) by a 2A sequence. (Scale bar, 10 µm.) (*C*) *Left* and *Right*: average whole-cell currents in response to voltage-clamp protocols (*Top*). *Middle*: steady-state activation (blue, *n* = 19) and inactivation curves (purple, *n* = 21) of S2R+ cells transfected with Sh isoform C. (*D*) *Left*: Confocal cross-section of the optic lobe with endogenously tagged Sh. Dashed lines, neuropil outlines based on Brp stainings. (Scale bar, 20 µm.) *Right*: Schematic of Sh localization (green) on the morphology of a single T5 neuron. (*E*) Steady-state activation (*n* = 6) and inactivation (*n* = 4) as in (*C*) for the coexpression of Sh and Hk. (*F*) Same as (*D*) but for Shab. (*G*) Steady-state activation (*n* = 15) and inactivation (*n* = 11) as in (*C*) for Shab isoform M. (*H*) Same as (*D*) but for Shal. (*I*) Steady-state activation (*n* = 12) and inactivation (*n* = 9) as in (*C*) for Shal isoform B. (*J*) Same as (*D*) but for Eag. (*K*) Steady-state activation (*n* = 11) and inactivation (*n* = 10) as in (*C*) for Eag isoform A.

To test this hypothesis, we used cell type–specific FlpTag to map ion channel localizations onto the well-defined morphologies of T4/T5 neurons (Fig. 3 and *SI Appendix*, Fig. S4*A*) and complemented this with a catalog of steady-state activation and inactivation curves of the respective channels. Since the unipolar anatomy and the thin neurites of these cells compromise voltage-clamp precision in situ, we resorted to an in vitro approach. We expressed the most abundant splice variant of each channel (Fig. 3*A*) (22) heterologously in *Drosophila* S2R+ cells (Fig. 3*B*) (47), whose voltage could be controlled with high fidelity in whole-cell patch-clamp experiments and whose plasma membranes, in their native state, were devoid of active conductances (*SI Appendix*, Fig. S4*B*).

Our experiments revealed a highly organized spatial segregation of potassium channels in T4/T5 neurons, and diverse gating functions in S2R+ cells. Sh, Shal, and—to a lesser extent—Shab were detectable in the axonal compartments and gave rise to outward currents of varying voltage dependencies and gating kinetics (Fig. 3 *C*–*I*). The activation and inactivation kinetics of Sh, which was also present in the dendritic compartment and highly enriched in axon terminals, could be further accelerated by coexpression with its cognate β subunit *Hyperkinetic* (*Hk*; Fig. 3*E* and *SI Appendix*, Fig. S4*C*) (48). Ether-à-go-go (Eag) (49), on the other hand, decorated the dendrites and had slower activation kinetics than its axonal counterparts (Fig. 3 *J* and *K*). Other candidate channels, like Shaker cognate w (Shaw) (39) and the predicted two-pore-domain channel *CG1688* were undetectable in T4/T5 neurons (*SI Appendix*, Fig. S4*D*) and yielded no measurable currents in S2R+ cells, while *Ih* channels were visible (*SI Appendix*, Fig. S4*D*) but functionally silent under our recording conditions.

### Localization and Function of Para Channels in T4 and T5 Neurons.

**Fig. 4.**
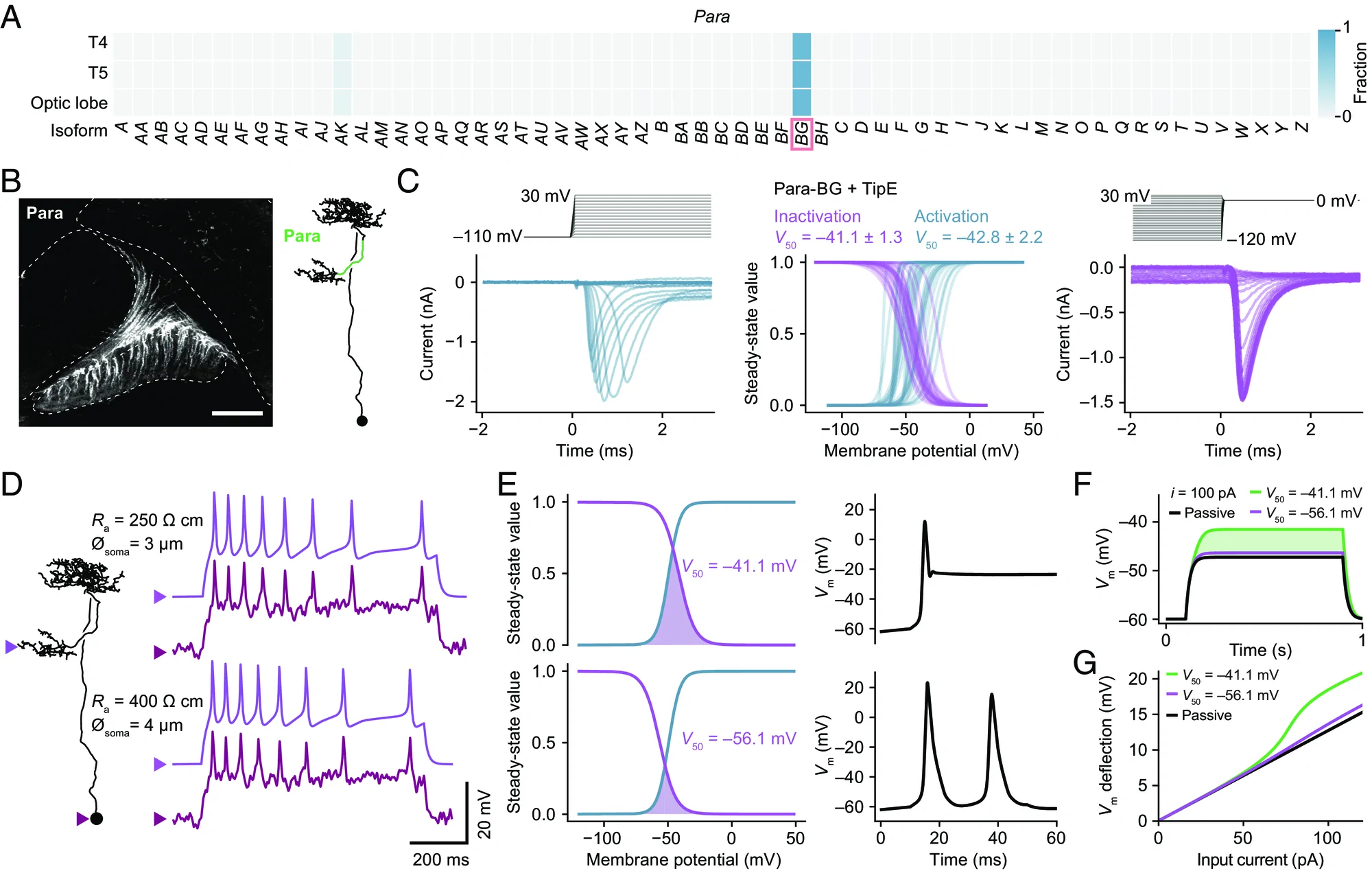
Supralinear signal amplification through sodium window currents. (*A*) Relative expression of Para splice variants in T4 and T5 neurons and the optic lobe. Data from Davis et al. (22). Isoform BG (red box) was cloned. (*B*) *Left*: Confocal cross-section of the optic lobe with tagged Para. Dashed lines, neuropil outlines based on Brp stainings. (Scale bar, 20 µm.) *Right*: Schematic of Para localization (green) on the morphology of a single T5 neuron. (*C*) *Left* and *Right*: average whole-cell currents of S2R+ cells transfected with Para in response to voltage steps (*Top*). *Middle*: steady-state activation (blue, *n* = 30) and inactivation curves (purple, *n* = 25) of Para isoform BG. (*D*) Spike amplitude attenuation in T5 neuron models with experimentally constrained potassium channels and conventional, “spiking” sodium channels confined to the axon. Input-evoked action potentials were detectable in the axon terminal (purple) and the soma (magenta) of two models with different axial resistivity (*R*_a_) and soma diameter (⌀_soma_). Triangles indicate recording sites. (*E*) *Left*: Para models with half-maximal inactivation (*V*_50_) as measured (*Top*) and shifted by –15 mV (*Bottom*). *Right*: Membrane potential (*V*_m_) responses to current injection into a model T5 neuron with native (*Top*) and shifted Para inactivation (*Bottom*). (*F*) Simulated *V*_m_ of the T5 neuron with passive membrane properties (black), with empirically constrained Para gating (green), and with shifted half-maximal Para inactivation (purple) in response to a square-wave current injection. Shaded area, supralinearity relative to the passive neuron. (*G*) Simulated *V*_m_ deflection as a function of input current for a T5 neuron with passive membrane properties (black), with experimentally constrained Para gating (green), and with shifted half-maximal Para inactivation (purple).

The voltage-gated sodium channel Para (isoform BG) clearly marked the axons but not the axon terminals of T4 and T5 neurons (Fig. 4 *A* and *B*). When coexpressed with its β subunit temperature-induced paralytic E (tipE) (50), Para produced fast inward currents with a curiously small separation between half-maximal activation (−41.1 ± 1.3 mV) and inactivation voltages (−42.8 ± 2.2 mV, Fig. 4*C*). This overlap came with a persistent Para current at membrane potentials between approximately −60 and −30 mV. “Window” currents like this arise over a range of voltages where the probability of activation is nonzero and steady-state inactivation is not complete (51). Under most conditions, they are small due to a clear separation of half-maximal activation and inactivation voltages, which amounts to 20 mV on average for different splice variants of Para (52). This keeps window currents small and favors rapid regenerative spiking. The unusual intersection point of Para gating functions observed here suggests an isoform-specific idiosyncrasy that might interfere with spike generation and propagation.

### Para Gating Functions Prevent Spiking in T4 and T5 Neurons.

To explore this possibility, we created a compartmental model of a T5 neuron that paid heed to the precise localizations and gating functions of Sh, Shab, Shal, and Eag (*SI Appendix*, Fig. S4 *E* and *F*) and systematically varied the biophysical properties of the axonal sodium conductance. When the model neuron contained a conventional sodium channel in place of Para, action potentials evoked by depolarizing dendritic current injection were readily detectable at the soma, even for extreme values of axial resistivity and soma diameter (Fig. 4*D*). T4 neurons, which exhibit larger variability in fiber lengths than their T5 relatives, showed similarly modest spike amplitude attenuation in examples of both short (*SI Appendix*, Fig. S5*A*) and particularly long neurites (*SI Appendix*, Fig. S5*B*). The presence of somatic spikes across all modeling regimes with conventional sodium channels is inconsistent with the reported lack of action potentials in electrophysiological recordings (44–46).

If, however, the axonal sodium conductance was tailored to match the empirically determined Para current, the T5 neuron failed to generate repeated action potentials in response to depolarizing current injection. Rescuing spiking activity in silico required a leftward shift of the Para inactivation curve by at least −15 mV (Fig. 4*E*). These simulations suggest that the inability to record action potentials from the somata of T4 and T5 neurons (44–46) is not a result of channel distribution or signal attenuation imposed by neuronal morphology but a consequence of specialized channel gating. This leaves us with a puzzle: with no spikes to generate, what function might Para serve in these cells?

### Persistent Sodium Currents Generate a Sustained Supralinearity.

T4 and T5 cells rely on nonlinearities when computing the direction of visual motion (53–55). Presynaptic interneurons differentially delay the signals from adjacent points in visual space (12, 56–59) enabling T4 and T5 neurons to read out the temporal sequence of photoreceptor activation by detecting the coincidence of two inputs. During motion in a neuron’s antipreferred direction, shunting inhibition cancels coincident inputs (45, 46, 60). Conversely, during motion in the preferred direction, coincident inputs are amplified supralinearly. In ON-selective T4 neurons, this enhancement is driven by multiplicative disinhibition (44, 61). In OFF-selective T5 neurons, however, the mechanistic basis of preferred direction enhancement (62) is elusive. It is unlikely to involve disinhibition, because T5 neurons lack the appropriate presynaptic partners (6, 21). We reasoned that the Para window current might provide a cell-intrinsic nonlinearity that amplifies coincident excitatory inputs, even in the absence of action potentials.

Model simulations confirmed this notion: the window current gave rise to a sustained supralinearity, meaning that the voltage deflection in response to depolarizing current injections exceeded the linear, passive membrane-based expectation (Fig. 4*F*). This amplification was tightly linked to the gating properties of Para and highly sensitive to changes in half-maximal activation and inactivation voltages. The customary −15-mV shift of the steady-state inactivation curve flattened the nonlinearity almost to the level of the passive membrane (Fig. 4*G*). Artificial manipulations of the Para window current, either through leftward shifts of the inactivation curve or rightward shifts of the activation curve, had graded effects on the nonlinearity (*SI Appendix*, Fig. S5*C*). Even though Para was confined to the axonal compartment (Fig. 4*B*), the supralinearity was also detectable in the axon terminal and, to a similar extent, in the soma (*SI Appendix*, Fig. S5*D*). This indicates that Para window currents provide a sustained nonlinearity, which might be well-suited to the computational needs of motion-sensitive graded-potential neurons.

### Axonal Para Currents Sharpen the Directional Tuning of Motion-Sensitive Neurons.

**Fig. 5.**
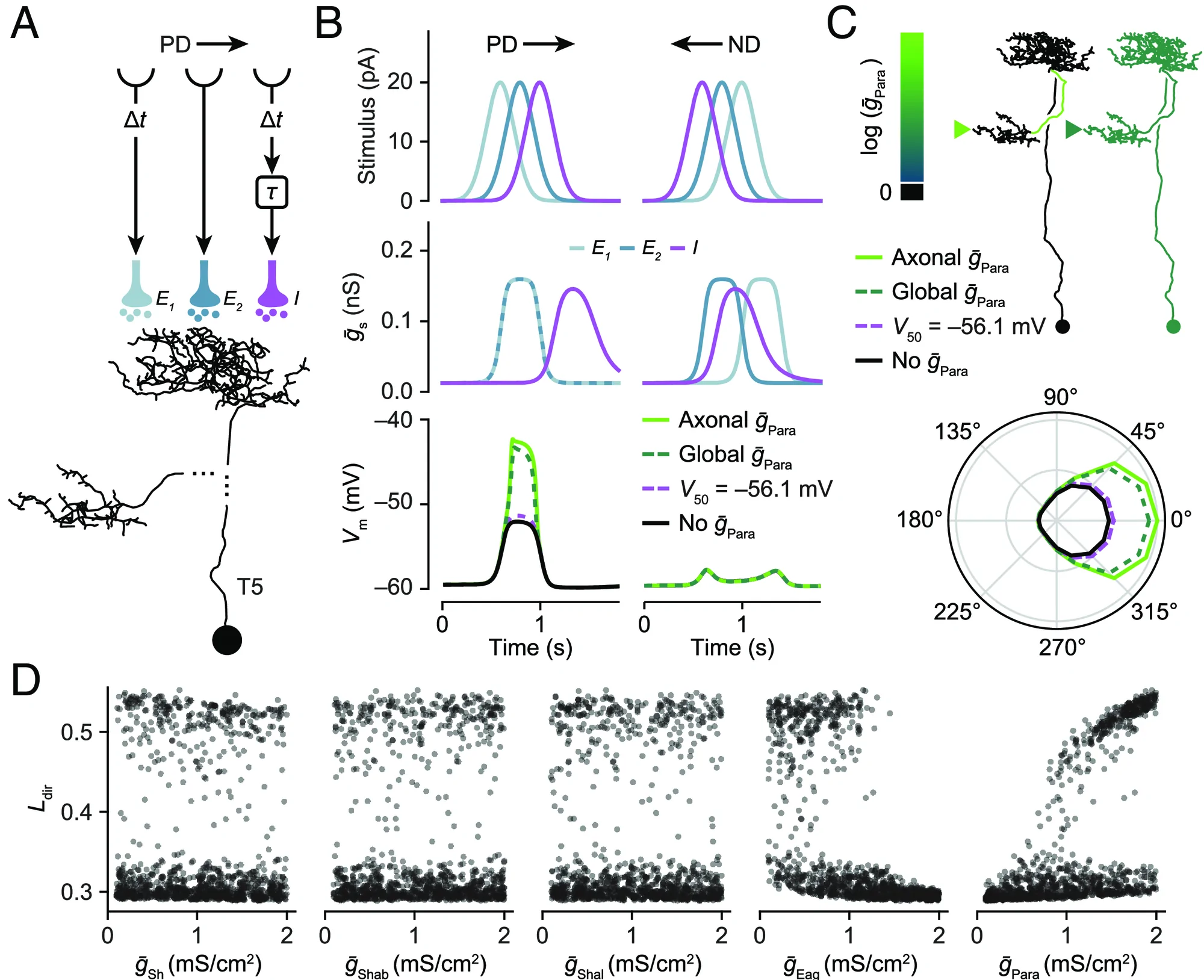
Channel density, distribution, and inactivation shape computation. (*A*) Experimentally constrained compartmental T5 circuit model. The three-arm motion detector received excitatory dendritic inputs (*E*_1_ and *E*_2_) from two points in visual space and inhibitory input (*I*) from a third. The signals *E*_1_ and *I* were delayed (Δ*t*) relative to *E*_2_, and *I* was low-pass filtered (*τ*). (*B*) Stimulus sequences (*Top*), synaptic conductances (*ḡ*_s_, *Center*), and membrane potential at the T5 axon terminal (*V*_m_, *Bottom*) with Para confined to the axon (light green), globally distributed Para (dashed dark green), axonal Para with a leftward shift in the inactivation curve (dashed purple), and without Para (black) for motion in the preferred (PD, *Left*) and in the null direction (ND, *Right*). (*C*) *Top*: Spatial distributions of Para conductance (*ḡ*_Para_) mapped onto the morphology of the T5 neuron. Triangles indicate recording site. *Bottom*: Normalized directional tuning curves of the model neuron with Para confined to the axon (light green), globally distributed Para (dashed dark green), axonal Para with a −15-mV shift in the inactivation curve (dashed purple), and without Para (black). For axonal and global distributions, total *ḡ*_Para_ was 246 pS/cell. (*D*) Directional tuning (*L*_dir_) of the model T5 neuron as a function of conductance density for 1,000 random combinations of *ḡ*_Sh_, *ḡ*_Shab_, *ḡ*_Shal_, *ḡ*_Eag_, and *ḡ*_Para_.

Nonlinearities are the defining features of two- and three-arm elementary motion detectors (53, 55, 63). To lend credibility to the hypothesis that Para window currents serve as a coincidence-detecting supralinearity in T5 neurons, we built a biologically constrained compartmental model. The model took into account the morphology of the T5 neuron—the same as before—and mapped onto it the subcellular localizations and empirically determined gating parameters of ion channels. The dendrite contained conductance-based synapses mimicking the spatial arrangement, temporal properties, and reversal potentials of those of the three main presynaptic partners of T5 neurons (21, 59, 60) (Fig. 5*A*). Varying temporal sequences of input currents corresponding to three adjacent points in visual space simulated a dark bar moving at 25°/s in 16 different directions (Fig. 5 *B* and *C*). As a readout, we constructed tuning curves based on the average membrane potential deflection of the T5 neuron during motion in the respective direction.

The constrained model produced highly direction-selective responses purely by the interplay of biophysical properties, without the need for artificial multiplicative or divisive nonlinearities (Fig. 5 *B* and *C*). The Para window current provided the required supralinearity. Closing the window through the customary −15-mV shift in half-maximal inactivation deteriorated the tuning curve and left the responses to preferred-direction motion unamplified. Global redistribution of Para channels across the T5 morphology had a similar, albeit less pronounced, effect (Fig. 5*C*).

To evaluate how variations in conductance densities of specific ion channels influence the direction selectivity of the model neuron, we performed a random parameter sweep across *ḡ*_Sh_, *ḡ*_Shab_, ḡ_Shal_, *ḡ*_Eag_, and ḡ_Para_. We generated a total of 1,000 distinct parameter combinations by uniformly sampling conductance densities from 0.1 to 2 mS/cm^2^ at their designated subcellular localizations. For each parameter profile, we calculated the resulting direction selectivity (*L*_dir_) of the T5 neuron based on the voltage responses to visual motion in different directions (Fig. 5*D* and *SI Appendix*, *Supporting Methods*). *L*_dir_ values were independent of *ḡ*_Sh_, *ḡ*_Shab_, and *ḡ*_Shal_ within the sampled range. For Eag and Para, however, not all conductance densities were compatible with sharp directional tuning. *L*_dir_ values greater than 0.5, commonly seen in direction-selective cells (44), were not observed with *ḡ*_Eag_ greater than 1.5 mS/cm^2^ and with *ḡ*_Para_ of less than 1.5 mS/cm^2^. While Eag is a known determinant of neuronal excitability (64), our results show that the persistent current of axonal Para channels provides a supralinearity that sharpens the directional tuning of nonspiking T5 neurons.

## Discussion

Just like real T5 neurons (46), our biophysical model—due to a peculiar constellation of sodium channel gating functions—does not produce action potentials, but uses the window current of axonal Para channels (Fig. 4) to compute the direction of visual motion (Fig. 5). In doing so, T5 cells seem to prioritize the richer information capacity of analog signals over the speed of a pulsatile code (65–67). Such graded transmission operates across a wide variety of both invertebrate and vertebrate neurons (68–74). Some of them employ a mixed analog/digital mode (74), which presents a tentative explanation for the presence of ion channels traditionally associated with spike generation in nonspiking neurons like T5. A shift in half-maximal activation or inactivation might have allowed evolution to repurpose the nonlinearities they offer from analog–digital conversion to more subtle computations. Tetrodotoxin-sensitive sodium channels, for example, have also been described in nonspiking interneurons of locusts, where, instead of spikes, they shape postsynaptic potentials (15).

Our simulations indicate that neural computations are sensitive to changes not only in channel gating, but also in subcellular channel distribution; even in small and compact T5 neurons. In large, compartmentalized neurons like the amacrine cell CT1 (75), the situation is different. Here the distribution of channels will likely play a more important role, similar to the spike time-defining influence it had in our unconstrained model neuron (Fig. 1 *A*–*C*). Strategically placed potassium channels could actively isolate compartments one from another, especially in situations where space imposes limitations on cable length. Conversely, in pyramidal cells of rodents—but not of humans (76)—localized voltage-dependent conductances and well-tuned receptor densities accentuate distal relative to proximal inputs to equalize the efficacy of distributed synapses (77, 78). Small neurons in the fly brain (7, 79, 80), and large but electrotonically compact neurons in the stomatogastric ganglion of the crab (81) instate similar forms of synaptic democracy even with predominantly passive membranes. Under these conditions, spatially confined conductances might provide an additional level of modulation (82).

Evaluating the functional consequences of channel distribution and gating, however, remains a major challenge for computational neuroscience. Virtually identical activity patterns can arise from completely disparate combinations of synaptic and active conductances across different compartments (83–85) (Fig. 5*D*). Synapse locations and weights can be approximated—with more or less precision—based on connectomic data (7, 86). Active properties and their distributions, however, cannot be inferred from ultrastructure alone, making additional constraints mandatory to understand how a given neuron actually computes.

So far, structural knowledge about subcellular channel distributions came either from the overexpression of tagged genes (17, 24, 36, 87, 88), from immunohistochemistry, or from the combination of constitutive endogenous tags with cell type–specific markers (89, 90). All of these approaches have limitations: cell type–specific overexpression driven by artificial promoters frequently causes protein aggregation in ectopic membranes (17, 24) (*SI Appendix*, Fig. S3). Antibodies and constitutive tags (89, 91), on the other hand, label gene products irrespective of cell type; and since neurites are inherently intertwined, the resulting images tend to lack clarity.

By conditionally labeling endogenous channels in genetically defined neurons, our FlpTag approach (Fig. 1) bypasses these problems in *Drosophila*. Together with isoform-specific voltage-clamp recordings (Figs. 3 and 4), it allows investigators to substitute arbitrary parameter optimization with cell type–specific spatial and functional data. Our resources are of broad relevance, because FlpTags are universally applicable, subcellular targeting is conserved (92–94), and the isoforms we characterized are found throughout the optic lobe (Figs. 3*A* and 4*A*) and the brain (22). We provide possibilities for grounding active compartmental models of fly neurons in biological reality. This could involve incorporating more biophysical detail into models (95) or simplifying them to retain only crucial functional properties, such as cell-intrinsic nonlinearities, while reducing computational costs.

Our simulations imply that precise channel gating and localization dictate computation. Direct experimental evidence, however, can only come from targeted genetic interventions that change native gating and hijack the intricate system that distributes channels to their distinct subcellular domains (93).

## Materials and Methods

### Multicompartment and Circuit Models.

Simulations were performed in Python v3.12 (Python Software Foundation) using the Jaxley library (18) and morphologies of a T5 (cell #703) and a T4 neuron (cell #1425307353) (21). Example neurons with long and short soma fibers were cell #101053 and cell #100158, respectively, from a whole-brain connectome (6). Model details are given in *SI Appendix*, *Supporting Methods*.

### Fly Husbandry.

Flies were raised at 25 °C and 60% humidity on a standard cornmeal medium under a 12 h–12 h light–dark cycle.

### Molecular Biology and Transgenesis.

Constructs containing ion channel α subunits and *EGFP*, or β subunits and *mCherry*, were cloned into an Ac5-STABLE2-neo backbone (47).

Constitutive-tag and FlpTag lines were generated using ΦC31 recombinase-mediated cassette exchange (27) to insert tags into intronic gene regions (*SI Appendix,* Table S1) using either a MiMIC (28) or a CRIMIC (29). Details are provided in *SI Appendix*, *Supporting Methods*.

### Immunohistochemistry.

Fly brains were dissected in phosphate-buffered saline (PBS) and fixed in PBS containing 4% (w/v) paraformaldehyde for 25 min at 22 °C. After fixation, brains were washed in PBS containing 0.3% (v/v) Triton X-100 (PBST), followed by blocking in 10% (v/v) normal goat serum (NGS) in 0.3% (v/v) PBST for at least 2 h at 22 °C. All antibody solutions were prepared in 0.3% PBST with 5% NGS. Primary and secondary antibodies were incubated at 4 °C for at least 48 h and washed four to five times for 30 min at room temperature between antibody applications and before mounting. Brains were mounted in SlowFade™ Gold Antifade Mountant (Thermo Fisher Scientific) and imaged using a Leica Stellaris confocal microscope. Antibodies and analysis details are provided in *SI Appendix*, *Supporting Methods*.

### Patch-Clamp Experiments.

Voltage clamp experiments were performed in *Drosophila* S2R+ cells at 22 to 23 °C using a MultiClamp 700B amplifier and a Digidata 1550B digitizer (Molecular Devices). Details concerning cell culture, solutions, experimental protocols, and analyses are provided in *SI Appendix*, *Supporting Methods*.

## Supplementary Material

Appendix 01 (PDF)

## Data Availability

.tiff, .pkl, .npy, .py, .ipynb, .csv, and .txt data have been deposited in Zenodo (https://doi.org/10.5281/zenodo.20752905). Previously published data were used for this work (21, 22).

## References

[1] Z. Zheng , A complete electron microscopy volume of the brain of adult *Drosophila melanogaster*. Cell **174**, 730–743.e22 (2018). 10.1016/j.cell.2018.06.019 PMC6063995 30033368

[2] L. K. Scheffer , A connectome and analysis of the adult *Drosophila* central brain. eLife **9**, e57443 (2020). 10.7554/eLife.57443 PMC7546738 32880371

[3] M. Winding , The connectome of an insect brain. Science **379**, eadd9330 (2023). 10.1126/science.add9330 PMC7614541 36893230

[4] S. Dorkenwald , Neuronal wiring diagram of an adult brain. Nature **634**, 124–138 (2024). 10.1038/s41586-024-07558-y PMC11446842 39358518

[5] A. Matsliah , Neuronal parts list and wiring diagram for a visual system. Nature **634**, 166–180 (2024). 10.1038/s41586-024-07981-1 PMC11446827 39358525

[6] A. Nern , Connectome-driven neural inventory of a complete visual system. Nature **641**, 1225–1237 (2025). 10.1038/s41586-025-08746-0 PMC12119369 40140576

[7] T. X. Liu, P. A. Davoudian, K. M. Lizbinski, J. M. Jeanne, Connectomic features underlying diverse synaptic connection strengths and subcellular computation. Curr. Biol. **32**, 559–569.e5 (2022). 10.1016/j.cub.2021.11.056 PMC8825683 34914905

[8] P. K. Shiu , A *Drosophila* computational brain model reveals sensorimotor processing. Nature **634**, 210–219 (2024). 10.1038/s41586-024-07763-9 PMC11446845 39358519

[9] J. K. Lappalainen , Connectome-constrained networks predict neural activity across the fly visual system. Nature **634**, 1132–1140 (2024). 10.1038/s41586-024-07939-3 PMC11525180 39261740

[10] J. Haag, A. Borst, Amplification of high-frequency synaptic inputs by active dendritic membrane processes. Nature **379**, 639–641 (1996).

[11] E. S. Fortune, G. J. Rose, Passive and active membrane properties contribute to the temporal filtering properties of midbrain neurons in vivo. J. Neurosci. **17**, 3815–3825 (1997). 10.1523/JNEUROSCI.17-10-03815.1997 PMC6573678 9133400

[12] A. Borst, Differential temporal filtering in the fly optic lobe. J. Comput. Neurosci. **53**, 507–520 (2025). 10.1007/s10827-025-00914-5 PMC12672718 41003956

[13] F. Gabbiani, H. G. Krapp, C. Koch, G. Laurent, Multiplicative computation in a visual neuron sensitive to looming. Nature **420**, 320–324 (2002). 10.1038/nature01190 12447440

[14] D. A. Hoffman, J. C. Magee, C. M. Colbert, D. Johnston, K^+^ channel regulation of signal propagation in dendrites of hippocampal pyramidal neurons. Nature **387**, 869–875 (1997). 10.1038/43119 9202119

[15] G. Laurent, Voltage-dependent nonlinearities in the membrane of locust nonspiking local interneurons, and their significance for synaptic integration. J. Neurosci. **10**, 2268–2280 (1990). 10.1523/JNEUROSCI.10-07-02268.1990 PMC6570372 2376774

[16] T. Branco , Near-perfect synaptic integration by Na_v_1.7 in hypothalamic neurons regulates body weight. Cell **165**, 1749–1761 (2016). 10.1016/j.cell.2016.05.019 PMC4912688 27315482

[17] L. N. Groschner, L. Chan Wah Hak, R. Bogacz, S. DasGupta, G. Miesenböck, Dendritic integration of sensory evidence in perceptual decision-making. Cell **173**, 894–905.e13 (2018). 10.1016/j.cell.2018.03.075 PMC5947940 29706545

[18] M. Deistler , Jaxley: Differentiable simulation enables large-scale training of detailed biophysical models of neural dynamics. Nat. Methods **22**, 2649–2657 (2025). 10.1038/s41592-025-02895-w PMC12695658 41233544

[19] Z. F. Mainen, T. J. Sejnowski, Influence of dendritic structure on firing pattern in model neocortical neurons. Nature **382**, 363–366 (1996). 10.1038/382363a0 8684467

[20] M. S. Maisak , A directional tuning map of *Drosophila* elementary motion detectors. Nature **500**, 212–216 (2013). 10.1038/nature12320 23925246

[21] K. Shinomiya , Comparisons between the ON- and OFF-edge motion pathways in the *Drosophila* brain. eLife **8**, e40025 (2019). 10.7554/eLife.40025 PMC6338461 30624205

[22] F. P. Davis , A genetic, genomic, and computational resource for exploring neural circuit function. eLife **9**, e50901 (2020). 10.7554/eLife.50901 PMC7034979 31939737

[23] J. T. Littleton, B. Ganetzky, Ion channels and synaptic organization. Neuron **26**, 35–43 (2000). 10.1016/s0896-6273(00)81135-6 10798390

[24] S. Fendl, R. M. Vieira, A. Borst, Conditional protein tagging methods reveal highly specific subcellular distribution of ion channels in motion-sensing neurons. eLife **9**, e62953 (2020). 10.7554/eLife.62953 PMC7655108 33079061

[25] P. Sanfilippo , Mapping of multiple neurotransmitter receptor subtypes and distinct protein complexes to the connectome. Neuron **112**, 942–958.e13 (2024). 10.1016/j.neuron.2023.12.014 PMC10957333 38262414

[26] S. Kondo , Neurochemical organization of the *Drosophila* brain visualized by endogenously tagged neurotransmitter receptors. Cell Rep. **30**, 284–297.e5 (2020). 10.1016/j.celrep.2019.12.018 31914394

[27] J. R. Bateman, A. M. Lee, C. Wu, Site-specific transformation of *Drosophila* via ϕC31 integrase-mediated cassette exchange. Genetics **173**, 769–777 (2006). 10.1534/genetics.106.056945 PMC1526508 16547094

[28] K. J. T. Venken , MiMIC: A highly versatile transposon insertion resource for engineering *Drosophila melanogaster* genes. Nat. Methods **8**, 737–743 (2011). 10.1038/nmeth.1662 PMC3191940 21985007

[29] O. Kanca , An expanded toolkit for *Drosophila* gene tagging using synthesized homology donor constructs for CRISPR-mediated homologous recombination. eLife **11**, e76077 (2022). 10.7554/eLife.76077 PMC9239680 35723254

[30] S. Viswanathan , High-performance probes for light and electron microscopy. Nat. Methods **12**, 568–576 (2015). 10.1038/nmeth.3365 PMC4573404 25915120

[31] T. Schlake, J. Bode, Use of mutated FLP recognition target (FRT) sites for the exchange of expression cassettes at defined chromosomal loci. Biochemistry **33**, 12746–12751 (1994). 10.1021/bi00209a003 7947678

[32] L. Salkoff, R. Wyman, Genetic modification of potassium channels in *Drosophila* Shaker mutants. Nature **293**, 228–230 (1981). 10.1038/293228a0 6268986

[33] B. Sauer, DNA recombination with a heterospecific Cre homolog identified from comparison of the pac-c1 regions of P1-related phages. Nucleic Acids Res. **32**, 6086–6095 (2004). 10.1093/nar/gkh941 PMC534624 15550568

[34] A. Nern, B. D. Pfeiffer, K. Svoboda, G. M. Rubin, Multiple new site-specific recombinases for use in manipulating animal genomes. Proc. Natl. Acad. Sci. U.S.A. **108**, 14198–14203 (2011). 10.1073/pnas.1111704108 PMC3161616 21831835

[35] B. Bausenwein, A. P. M. Dittrich, K.-F. Fischbach, The optic lobe of *Drosophila melanogaster*: II. Sorting of retinotopic pathways in the medulla. Cell Tissue Res. **267**, 17–28 (1992). 10.1007/BF00318687 1735111

[36] F. Kawasaki, B. Zou, X. Xu, R. W. Ordway, Active zone localization of presynaptic calcium channels encoded by the cacophony locus of *Drosophila*. J. Neurosci. **24**, 282–285 (2004). 10.1523/JNEUROSCI.3553-03.2004 PMC6729574 14715960

[37] F. Von Schilcher, The behavior of cacophony, a courtship song mutant in *Drosophila melanogaster*. Behav. Biol. **17**, 187–196 (1976). 10.1016/s0091-6773(76)90444-2 822818

[38] L. A. Smith , A *Drosophila* calcium channel α1 subunit gene maps to a genetic locus associated with behavioral and visual defects. J. Neurosci. **16**, 7868–7879 (1996). 10.1523/JNEUROSCI.16-24-07868.1996 PMC6579206 8987815

[39] A. Butler, A. Wei, K. Baker, L. Salkoff, A family of putative potassium channel genes in *Drosophila*. Science **243**, 943–947 (1989). 10.1126/science.2493160 2493160

[40] O. Siddiqi, S. Benzer, Neurophysiological defects in temperature-sensitive paralytic mutants of *Drosophila melanogaster*. Proc. Natl. Acad. Sci. U.S.A. **73**, 3253–3257 (1976). 10.1073/pnas.73.9.3253 PMC430996 184469

[41] K. Loughney, R. Kreber, B. Ganetzky, Molecular analysis of the para locus, a sodium channel gene in *Drosophila*. Cell **58**, 1143–1154 (1989). 10.1016/0092-8674(89)90512-6 2550145

[42] A. L. Hodgkin, A. F. Huxley, A quantitative description of membrane current and its application to conduction and excitation in nerve. J. Physiol. **117**, 500–544 (1952). 10.1113/jphysiol.1952.sp004764 PMC1392413 12991237

[43] K. Shinomiya , The organization of the second optic chiasm of the *Drosophila* optic lobe. Front. Neural Circuits **13**, 65 (2019). 10.3389/fncir.2019.00065 PMC6797552 31680879

[44] L. N. Groschner, J. G. Malis, B. Zuidinga, A. Borst, A biophysical account of multiplication by a single neuron. Nature **603**, 119–123 (2022). 10.1038/s41586-022-04428-3 PMC8891015 35197635

[45] E. Gruntman, S. Romani, M. B. Reiser, Simple integration of fast excitation and offset, delayed inhibition computes directional selectivity in *Drosophila*. Nat. Neurosci. **21**, 250–257 (2018). 10.1038/s41593-017-0046-4 PMC5967973 29311742

[46] E. Gruntman, S. Romani, M. B. Reiser, The computation of directional selectivity in the *Drosophila* OFF motion pathway. eLife **8**, e50706 (2019). 10.7554/eLife.50706 PMC6917495 31825313

[47] M. González , Generation of stable *Drosophila* cell lines using multicistronic vectors. Sci. Rep. **1**, 75 (2011). 10.1038/srep00075 PMC3216562 22355594

[48] S. W. Chouinard, G. F. Wilson, A. K. Schlimgen, B. Ganetzky, A potassium channel beta subunit related to the aldo-keto reductase superfamily is encoded by the *Drosophila* hyperkinetic locus. Proc. Natl. Acad. Sci. U.S.A. **92**, 6763–6767 (1995). 10.1073/pnas.92.15.6763 PMC41409 7542775

[49] J. Warmke, R. Drysdale, B. Ganetzky, A distinct potassium channel polypeptide encoded by the *Drosophila* eag locus. Science **252**, 1560–1562 (1991). 10.1126/science.1840699 1840699

[50] G. Feng, P. Deak, M. Chopra, L. M. Hall, Cloning and functional analysis of tipE, a novel membrane protein that enhances *Drosophila* para sodium channel function. Cell **82**, 1001–1011 (1995). 10.1016/0092-8674(95)90279-1 7553842

[51] D. Attwell, I. Cohen, D. Eisner, M. Ohba, C. Ojeda, The steady state TTX-sensitive (“window”) sodium current in cardiac Purkinje fibres. Pflugers Arch. **379**, 137–142 (1979). 10.1007/BF00586939 571107

[52] R. O. Olson, Z. Liu, Y. Nomura, W. Song, K. Dong, Molecular and functional characterization of voltage-gated sodium channel variants from *Drosophila melanogaster*. Insect Biochem. Mol. Biol. **38**, 604–610 (2008). 10.1016/j.ibmb.2008.01.003 PMC3056540 18405837

[53] J. Haag, A. Arenz, E. Serbe, F. Gabbiani, A. Borst, Complementary mechanisms create direction selectivity in the fly. eLife **5**, e17421 (2016). 10.7554/eLife.17421 PMC4978522 27502554

[54] J. C. S. Leong, J. J. Esch, B. Poole, S. Ganguli, T. R. Clandinin, Direction selectivity in *Drosophila* emerges from preferred-direction enhancement and null-direction suppression. J. Neurosci. **36**, 8078–8092 (2016). 10.1523/JNEUROSCI.1272-16.2016 PMC4971360 27488629

[55] A. Borst, L. N. Groschner, How flies see motion. Annu. Rev. Neurosci. **46**, 17–37 (2023). 10.1146/annurev-neuro-080422-111929 37428604

[56] G. Ammer, A. Leonhardt, A. Bahl, B. J. Dickson, A. Borst, Functional specialization of neural input elements to the *Drosophila* ON motion detector. Curr. Biol. **25**, 2247–2253 (2015). 10.1016/j.cub.2015.07.014 26234212

[57] J. A. Strother , The emergence of directional selectivity in the visual motion pathway of *Drosophila*. Neuron **94**, 168–182.e10 (2017). 10.1016/j.neuron.2017.03.010 28384470

[58] A. Arenz, M. S. Drews, F. G. Richter, G. Ammer, A. Borst, The temporal tuning of the *Drosophila* motion detectors is determined by the dynamics of their input elements. Curr. Biol. **27**, 929–944 (2017). 10.1016/j.cub.2017.01.051 28343964

[59] E. Serbe, M. Meier, A. Leonhardt, A. Borst, Comprehensive characterization of the major presynaptic elements to the *Drosophila* OFF motion detector. Neuron **89**, 829–841 (2016). 10.1016/j.neuron.2016.01.006 26853306

[60] A. Braun, A. Borst, M. Meier, Disynaptic inhibition shapes tuning of OFF-motion detectors in *Drosophila*. Curr. Biol. **33**, 2260–2269.e4 (2023). 10.1016/j.cub.2023.05.007 37236181

[61] A. Borst, A biophysical mechanism for preferred direction enhancement in fly motion vision. PLoS Comput. Biol. **14**, e1006240 (2018). 10.1371/journal.pcbi.1006240 PMC6016951 29897917

[62] J. Haag, A. Mishra, A. Borst, A common directional tuning mechanism of *Drosophila* motion-sensing neurons in the ON and in the OFF pathway. eLife **6**, e29044 (2017). 10.7554/eLife.29044 PMC5582866 28829040

[63] B. Hassenstein, W. Reichardt, Systemtheoretische Analyse der Zeit-, Reihenfolgen- und Vorzeichenauswertung bei der Bewegungsperzeption des Rüsselkäfers *Chlorophanus*. Z. Naturforsch. B **11**, 513–524 (1956).

[64] B. Ganetzky, G. A. Robertson, G. F. Wilson, M. C. Trudeau, S. A. Titus, The eag family of K^+^ channels in *Drosophila* and mammals. Ann. N. Y. Acad. Sci. **868**, 356–369 (1999). 10.1111/j.1749-6632.1999.tb11297.x 10414305

[65] R. R. de Ruyter van Steveninck, S. B. Laughlin, The rate of information transfer at graded-potential synapses. Nature **379**, 642–645 (1996).

[66] J. Haag, A. Borst, Active membrane properties and signal encoding in graded potential neurons. J. Neurosci. **18**, 7972–7986 (1998). 10.1523/JNEUROSCI.18-19-07972.1998 PMC6793010 9742164

[67] R. A. DiCaprio, Information transfer rate of nonspiking afferent neurons in the crab. J. Neurophysiol. **92**, 302–310 (2004). 10.1152/jn.01176.2003 14973322

[68] F. S. Werblin, J. E. Dowling, Organization of the retina of the mudpuppy, *Necturus maculosus*. II. Intracellular recording. J. Neurophysiol. **32**, 339–355 (1969). 10.1152/jn.1969.32.3.339 4306897

[69] M. Mendelson, Oscillator neurons in crustacean ganglia. Science **171**, 1170–1173 (1971). 10.1126/science.171.3976.1170 5544877

[70] K. G. Pearson, C. R. Fourtner, Nonspiking interneurons in walking system of the cockroach. J. Neurophysiol. **38**, 33–52 (1975). 10.1152/jn.1975.38.1.33 162945

[71] D. M. Maynard, K. D. Walton, Effects of maintained depolarization of presynaptic neurons on inhibitory transmission in lobster neuropil. J. Comp. Physiol. **97**, 215–243 (1975).

[72] M. Burrows, M. V. S. Siegler, Transmission without spikes between locust interneurones and motoneurones. Nature **262**, 222–224 (1976). 10.1038/262222a0 180435

[73] K. Graubard, Synaptic transmission without action potentials: Input-output properties of a nonspiking presynaptic neuron. J. Neurophysiol. **41**, 1014–1025 (1978). 10.1152/jn.1978.41.4.1014 210264

[74] K. Graubard, J. A. Raper, D. K. Hartline, Graded synaptic transmission between spiking neurons. Proc. Natl. Acad. Sci. U.S.A. **77**, 3733–3735 (1980). 10.1073/pnas.77.6.3733 PMC349693 6106194

[75] M. Meier, A. Borst, Extreme compartmentalization in a *Drosophila* amacrine cell. Curr. Biol. **29**, 1545–1550.e2 (2019). 10.1016/j.cub.2019.03.070 31031119

[76] L. Beaulieu-Laroche , Enhanced dendritic compartmentalization in human cortical neurons. Cell **175**, 643–651.e14 (2018). 10.1016/j.cell.2018.08.045 PMC6197488 30340039

[77] M. Häusser, Synaptic function: Dendritic democracy. Curr. Biol. **11**, R10–R12 (2001). 10.1016/s0960-9822(00)00034-8 11166188

[78] M. Häusser, N. Spruston, G. J. Stuart, Diversity and dynamics of dendritic signaling. Science **290**, 739–744 (2000). 10.1126/science.290.5492.739 11052929

[79] A. Moreno-Sanchez , Morphology and synapse topography optimize linear encoding of synapse numbers in *Drosophila* looming responsive descending neurons. eLife **13**, RP99277 (2024).

[80] O. A. Hafez , The cellular architecture of memory modules in *Drosophila* supports stochastic input integration. eLife **12**, e77578 (2023). 10.7554/eLife.77578 PMC10069864 36916672

[81] A. G. Otopalik, A. C. Sutton, M. Banghart, E. Marder, When complex neuronal structures may not matter. eLife **6**, e23508 (2017). 10.7554/eLife.23508 PMC5323043 28165322

[82] A. Takashima, M. Takahata, Effects of active conductance distribution over dendrites on the synaptic integration in an identified nonspiking interneuron. PLoS ONE **3**, e2217 (2008). 10.1371/journal.pone.0002217 PMC2375052 18493322

[83] A. A. Prinz, D. Bucher, E. Marder, Similar network activity from disparate circuit parameters. Nat. Neurosci. **7**, 1345–1352 (2004). 10.1038/nn1352 15558066

[84] A. L. Taylor, J.-M. Goaillard, E. Marder, How multiple conductances determine electrophysiological properties in a multicompartment model. J. Neurosci. **29**, 5573–5586 (2009). 10.1523/JNEUROSCI.4438-08.2009 PMC2821064 19403824

[85] L. M. Alonso, E. Marder, Visualization of currents in neural models with similar behavior and different conductance densities. eLife **8**, e42722 (2019). 10.7554/eLife.42722 PMC6395073 30702427

[86] S. Holler, G. Köstinger, K. A. C. Martin, G. F. P. Schuhknecht, K. J. Stratford, Structure and function of a neocortical synapse. Nature **591**, 111–116 (2021). 10.1038/s41586-020-03134-2 33442056

[87] S. Trunova, B. Baek, E. Giniger, Cdk5 regulates the size of an axon initial segment-like compartment in mushroom body neurons of the *Drosophila* central brain. J. Neurosci. **31**, 10451–10462 (2011). 10.1523/JNEUROSCI.0117-11.2011 PMC3150738 21775591

[88] Y. Ping , Shal/K_v_4 channels are required for maintaining excitability during repetitive firing and normal locomotion in *Drosophila*. PLoS ONE **6**, e16043 (2011). 10.1371/journal.pone.0016043 PMC3022017 21264215

[89] T. A. Ravenscroft , *Drosophila* voltage-gated sodium channels are only expressed in active neurons and are localized to distal axonal initial segment-like domains. J. Neurosci. **40**, 7999–8024 (2020). 10.1523/JNEUROSCI.0142-20.2020 PMC7574647 32928889

[90] J. Werner, J. Arian, I. Bernhardt, S. Ryglewski, C. Duch, Differential localization of voltage-gated potassium channels during *Drosophila* metamorphosis. J. Neurogenet. **34**, 133–150 (2020). 10.1080/01677063.2020.1715972 31997675

[91] S. Rey , Glial-dependent clustering of voltage-gated ion channels in *Drosophila* precedes myelin formation. eLife **12**, e85752 (2023). 10.7554/eLife.85752 PMC10287160 37278291

[92] C. Gu, Y. N. Jan, L. Y. Jan, A conserved domain in axonal targeting of K_v_1 (Shaker) voltage-gated potassium channels. Science **301**, 646–649 (2003). 10.1126/science.1086998 12893943

[93] H. C. Lai, L. Y. Jan, The distribution and targeting of neuronal voltage-gated ion channels. Nat. Rev. Neurosci. **7**, 548–562 (2006). 10.1038/nrn1938 16791144

[94] J. F. Rivera, S. Ahmad, M. W. Quick, E. R. Liman, D. B. Arnold, An evolutionarily conserved dileucine motif in Shal K^+^ channels mediates dendritic targeting. Nat. Neurosci. **6**, 243–250 (2003). 10.1038/nn1020 12592409

[95] W. F. Podlaski , Mapping the function of neuronal ion channels in model and experiment. eLife **6**, e22152 (2017). 10.7554/eLife.22152 PMC5340531 28267430

