# Supplementary material for: Distribution and voltage dependence of ion channels shape single-neuron computations
Source: Proc Natl Acad Sci U S A. 2026 Sep 29;123(40):e2610600123. doi: 10.1073/pnas.2610600123 (PMC13643278; doi:10.1073/pnas.2610600123)
Supplement: Supplementary file 1 [file pnas.2610600123.sapp.pdf]

Supporting Information for

Distribution and voltage-dependence of ion channels shape single-neuron computations

Renée M. Vieira, Michael Deistler, Clémentine Guillemet, Tabea Schilling, Jakob H. Macke, Alexander Borst, and Lukas N. Groschner

Lukas N. Groschner
Email: l.groschner@medunigraz.at

This PDF file includes:

Supporting Methods
Figures S1 to S5
Tables S1 to S2
SI References

Supporting Methods

Multi-compartment models

Simulations were performed in Python v3.12 (Python Software Foundation) using the Jaxley library (1). The morphologies of the T4 neuron (cell #1425307353) and the T5 neuron (cell #703) (2) were discretized into compartments using the $d\lambda$ rule (3) with $d\lambda = 0.1$, which resulted in 320 and 430 compartments, respectively. The surface area of the model matched the surface area of the respective SWC file. The example neurons with long and short soma fibers were taken from a whole-brain connectome (cell #101053 and cell #100158, respectively) (4) and consisted of 75 and 73 compartments, respectively. Because somata were not contained in the electron microscopic reconstructions, unless stated otherwise, a soma with a diameter of 2.6 μm was added to each of the compartmental model neurons. We assumed a specific membrane resistance (R_m) of 20 kΩ·cm^2, an axial resistivity (R_a) of 250 Ω·cm and a specific membrane capacitance of 1 μF/cm^2. These are standard values for invertebrate neurons (5–7) and consistent with input resistance measurements from T4 neurons (8). All simulations were performed with a time step of 25 μs. For visualization purposes only, axon and axon terminals of the T5 neuron morphology were laterally transposed by 5 μm in the graphics.

The Hodgkin-Huxley-type model (Fig. 1 *A*–*C*) included a passive leak conductance (g_{leak} = 25 μS/cm^2) and the default active sodium and potassium conductances implemented in Jaxley (9, 1). A fluctuating current stimulus generated using Gaussian-based sampling was injected at 32 distinct dendritic branches. The membrane potential was recorded at the soma and the axon terminal. We compared three channel distributions: 1) Sodium channels were localized to the axon (g_{Na} = 30 mS/cm^2), while leak and potassium channels were distributed homogeneously (Fig. 1*A*). 2) Sodium channels were distributed across the entire cell, with g_{Na} scaled to maintain the same total conductance per cell (Fig. 1*B*). 3) A uniformly distributed g_{Na} was determined by performing a parameter sweep with grid spacing of 0.1 mS/cm^2 to find the value that best approximated the response observed in the localized axonal model (Fig. 1*C*).

To probe action potential propagation, depolarizing step currents were applied to 32 dendritic locations. To mimic experimental recording conditions, which might complicate spike detection, in Fig. 4*D* and *SI Appendix*, Fig. S5 *A* and *B*, correlated observational noise was added by convolving a white noise signal with a Gaussian kernel (σ = 2.5 ms). The neuron contained voltage-gated sodium channels localized to the axon and a set of potassium channels, whose gating functions and localizations were experimentally constrained (Fig. 3 and *SI Appendix*, Fig. S4 *E* and *F*). Simulations were run in extreme morphological variants featuring a long (cell #101053) and a short soma fiber (cell #100158) (4). To assess the robustness of signal attenuation two biophysical regimes were tested for each cell: a high-resistance regime with R_a = 400 Ω·cm and a soma diameter of 4 μm and a low-resistance regime with R_a = 250 Ω·cm and a soma diameter of 3 μm (*SI Appendix*, Fig. S5 *A* and *B*).

To quantify supralinear response components, we compared voltage responses to current injections of active models to that of a passive model. Supralinearity was defined as the positive deflection of the active membrane potential response above the passive expectation.

Circuit model

Two excitatory and one inhibitory presynaptic cell were modeled as point neurons and connected through synapses to distinct dendritic locations of the compartmental T5 neuron (cell #703) (2). Synaptic conductance ($\bar{g}_s$) was modeled as a function of presynaptic membrane potential (V_m) using the transfer function

$$\bar{g}_s(V_m) = \frac{1}{1 + e^{-\frac{V_m - V_t}{k}}} \;;$$

with a threshold voltage (V_t) of –55 mV and a slope (k) of 2 mV. Reversal potentials were set to 0 mV for excitatory synapses and –60 mV for the inhibitory synapse.

Moving dark bars were simulated by injecting Gaussian current pulses into presynaptic neurons. The temporal sequence in which presynaptic neurons were stimulated mimicked a sweep at a velocity (v) of 25°/s. For a given motion angle φ, the delay between inputs was calculated as $\Delta t = \theta \cdot \cos\varphi / v$ assuming an inter-ommatidial angle θ of 5°. To construct a three-arm motion detector (8, 10, 11), a delay of 200 ms was added to the first and third input arms. Signals of the inhibitory presynaptic neuron were additionally low-pass filtered by increasing the membrane capacitance. The network was tested with motion in 16 different directions separated by 22.5° and directional tuning was quantified by calculating the mean membrane potential deflection at the axon terminal relative to the resting membrane potential. Results were normalized to the maximum response of the model with axonal Para.

To evaluate how variations in ion channel conductance densities influence the direction selectivity of the model T5 neuron, we performed a random parameter sweep across $\bar{g}_{Sh}$, $\bar{g}_{Shab}$, $\bar{g}_{Shal}$, $\bar{g}_{Eag}$, and $\bar{g}_{Para}$. A total of 1,000 distinct parameter combinations were generated by uniformly sampling conductance densities from 0.1 to 2 mS/cm^2. For each parameter profile, the multicompartmental network activity was simulated the 16 distinct directions of visual motion. For each motion angle φ, the response $|\mathbf{r}(\varphi)|$ was defined as the time-averaged membrane potential relative to baseline, normalized to the maximum response across all directions. The magnitude of direction selectivity (L_{dir}) was calculated as the length of the normalized circular variance vector:

$$L_{dir} = \left| \frac{\Sigma_{\varphi} \boldsymbol{r}(\varphi)}{\Sigma_{\varphi} |\boldsymbol{r}(\varphi)|} \right|$$

Transgenesis

Constitutive-tag and FlpTag lines were generated using ΦC31 recombinase-mediated cassette exchange (12) to insert tags into intronic gene regions (Table S1) using either a *Minos*-mediated integration cassette (MiMIC) (13) or a CRISPR-mediated integration cassette (CRIMIC) (14). Embryo injections were carried out by BestGene Inc. (Chino Hills, CA). Due to the non-directional nature of ΦC31 recombinase-mediated cassette exchange, FlpTag lines were generated in both the conditional and the constitutive orientation. The orientation of each transgenic line was confirmed by polymerase chain reaction. Lines carrying cassettes both the conditional and the constitutive orientation were utilized in experiments as indicated.

Cell type specificity for T4/T5 neurons was achieved by crossing FlpTag lines to flies carrying *R42F06-GAL4* (BL# 41253) and *UAS-FLP1.D* (BL# 4539). *R42F06-GAL4* targeted *GAL4* expression to T4/T5 neurons, where it activated transcription of *UAS-FLP1.D*. For experiments requiring additional cellular labeling, *UAS-myr::tdTomato* (BL# 32222) was used.

Stochastic labeling of single T4/T5 neurons in combination with FlpTag ion channel tagging was achieved through two different recombinase-based approaches. The first was a Dre-Flp dual recombinase system for constant sparse labeling. Since T4/T5 neurons are numerous, the Dre-rox system consistently generated individual tagged cells. *R42F06-GAL4* drove the expression of *3×UAS-Dre::PEST*. The Dre-recombinase induced low-efficiency excision of a stop cassette, enabling expression of Flp recombinase under *UAS* control. Flp recombinase then catalyzed the highly efficient flipping of *FRT* sites in the FlpTag cassette, resulting in switching the ion channel tag from a non-functional to a functional configuration. Simultaneously, *UAS-FRT-STOP-FRT-CD4::tdTomato* served to label the neurons.

The second system employed a titratable approach using heat shock-induced sparse labeling with the KD-Flp recombinase system. In this method, the *hsp70-KD::PEST* construct allowed for the excision of a stop cassette tunable by heat-shock. The sparse expression of Flp recombinase mediated the efficient flipping of the FRT sites within the FlpTag construct (*SI Appendix*, Fig. S2*C*), switching the ion channel tag from a non-functional to a functional configuration. *UAS-FRT-STOP-FRT-CD4::tdTomato* expression provided a visual marker for targeted neurons. To induce *KD::PEST* expression, pupae (P1–P3) were heat-shocked in a water bath at 37°C for 60–90 seconds.

Molecular biology

FlpTag-GFP constructs were generated as previously described (15). To create *FlpTag-3×V5*, the *FlpTag-GFP-ph0* construct was digested with EcoRI and HindIII (New England Biolabs). Three different 3×V5 constructs corresponding to the three intronic phases were synthesized by Eurofins, each containing a flexible 4×Gly–Gly–Ser linker and three V5 epitopes flanked by EcoRI and HindIII restriction sites. These constructs were inserted into the digested *FlpTag-GFP-ph0* plasmid via restriction cloning. Additionally, BamHI sites were incorporated immediately after the first and last Gly–Gly–Ser linker to facilitate the exchange of the 3×V5 tag with other visual markers. FlpTag-smV5 constructs were generated by digesting each of the three FlpTag-3×V5 phases and incorporating a synthesized smV5 tag flanked by BamHI sites. The smV5 tag was synthesized by Genewiz (Azenta).

The *UAS-rox-dSTOP-rox-Flp* plasmid was generated based on *pJFRC176-10XUAS-rox>-dSTOP-rox>-myr::GFP*. The *GFP* sequence was removed by digesting the plasmid with the restriction enzymes XhoI and XbaI (New England Biolabs). A synthetic *Flp* sequence, designed with flanking XhoI and XbaI restriction sites, was synthesized using the GeneArt Strings DNA Fragments (Invitrogen). This synthetic Flp fragment was inserted into the digested plasmid backbone using standard restriction cloning. Similarly, the *UAS-KDRT-dSTOP-KDRT-Flp* plasmid was generated by modifying *pJFRC164-21XUAS-KDRT>-dSTOP-KDRT>-myr::RFP* using the same approach described above.

To construct the *hsp70-IVS-KD::PEST* plasmid, we modified *pJFRC161-20XUAS-IVS-KD::PEST* (addgene plasmid #32140). The *20×UAS* sequence upstream of the hsp70 promoter was removed by digesting the plasmid with HindIII and AatII restriction enzymes. The ends were blunted using the Quick Blunting Kit (New England Biolabs, E1201) and ligated using the Quick Ligation Kit (New England Biolabs, M2200).

Constructs containing ion channel α subunits and *EGFP*, or β subunits and *mCherry*, were cloned into an Ac5-STABLE2-neo backbone (16) (addgene #32426). The most highly expressed splice variant of each ion channel gene was identified in a published RNA-sequencing dataset (17) using custom-written code in Python v.3.13. The coding sequence (CDS) of the respective isoform was retrieved from FlyBase and modified to exclude the start and stop codons and to include an EcoRI restriction site (5'–GAATTC–3') at the 5' end, and a NotI restriction site (5'–GCGGCCGC–3') in phase G at the 3' end. A Kozak sequence (5'–GCCACC–3') and a start codon (ATG) were added upstream of the CDS to ensure efficient translation. Sequences were synthesized by Genewiz (Azenta) and inserted into the Ac5-STABLE2-neo vector using EcoRI and NotI restriction sites. The same procedure was followed for generating plasmids expressing ion channel β subunits and mCherry, except that the constructs were synthesized with XbaI and HindIII restriction sites and the vector was digested using XbaI and HindIII. When co-expressing two plasmids containing an α subunit and a β subunit, the linked expression of *EGFP* and *mCherry* allowed to confirm effective co-transfection under the microscope.

Immunohistochemistry

Fly brains were dissected in phosphate-buffered saline (PBS; 137 mM NaCl, 3 mM KCl, 8 mM Na_2HPO_4, 1.5 mM KH_2PO_4, pH 7.3) and fixed in PBS containing 4% (w/v) paraformaldehyde for 25 minutes at 22 °C. After fixation, brains were washed four to five times for 30 minutes with PBS containing 0.3% (v/v) Triton X-100 (PBST), followed by blocking in 10% (v/v) normal goat serum (NGS) in 0.3% (v/v) PBST for at least 2 hours at 22 °C.

For immunohistochemistry, the following primary antibodies were used: mouse anti-bruchpilot (nc82, AB_2314866, Developmental Studies Hybridoma Bank, 1:20) for labelling synapses and chicken anti-V5 (AB_66741, Bethyl Laboratories, 1:500) for detecting V5-tagged proteins and rabbit anti-dsRed (AB_10013483, Takara Bio, 1:300,) for tdTomato-labelled neurons. Secondary antibodies were goat anti-mouse Alexa Fluor 488 (AB_2534069, Thermo Fisher Scientific, 1:500), goat anti-mouse Alexa Fluor 568 (AB_2534072, Thermo Fisher Scientific, 1:500), goat anti-chicken Alexa Fluor Plus 647 (AB_2762845, Thermo Fisher Scientific, 1:500), and goat anti-rabbit Alexa Fluor 568 (AB_10563601, Thermo Fisher Scientific, 1:500) for tdTomato-labeled neurons.

In cases where GFP was imaged, an Atto 647N-conjugated GFP booster (gba647n, ChromoTek, 1:500) was used. All antibody solutions were prepared in 0.3% PBST with 5% NGS. Primary and secondary antibodies were incubated at 4°C for at least 48 hours and washed four to five times for 30 minutes at room temperature between antibody applications and before mounting.

Imaging

Following immunostainings, brains were mounted in SlowFade™ Gold Antifade Mountant (Thermo Fisher Scientific) and imaged using a Leica Stellaris confocal microscope equipped with a white-light laser with wavelengths of 440–790 nm. Whole-brain mounts were imaged with a 20× glycerol-immersion objective; detailed images of optic lobes were taken using a HCX PL APO ×63/1.30 NA glycerol immersion objective (506353, Leica). Micrographs were acquired using the Leica Application Suite X (Leica) and processed using the Fiji distribution of ImageJ (v.2.0).

Clustering of spatial distributions

Fluorescence intensity profiles were measured along line profiles of the columnar axis of the medulla, lobula, lobula plate, and a single region of interest encompassing the inner chiasm. To account for the biological variability in neuropil dimensions, spatial intensity profiles within each region were standardized and linearly interpolated to match the maximum length observed for that specific region, effectively mapping all profiles onto a normalized distance scale from 0 to 1. To reduce high-frequency noise while preserving spatial distributions, a moving average filter was applied to the interpolated profiles using a centered window of 15 points. Intensity profiles were first averaged across samples for each ion channel and anatomical region and then divided by the maximum intensity of each ion channel across all regions.

To identify similarities in the patterns of spatial distributions of different ion channels across the entire visual system, a global intensity matrix was constructed where rows represented individual ion channels and columns represented the mean normalized intensities across all standardized points and anatomical regions. Hierarchical clustering was performed using Ward’s variance minimization method, and dissimilarity between expression profiles was calculated based on Euclidean distance. The resulting linkage matrix was used to plot a dendrogram.

Cell culture

Drosophila S2R+ cells were obtained from the Drosophila Genomics Resource Center (Bloomington, IN) and cultured at 25˚C in Schneider's *Drosophila* medium supplemented with 1% penicillin-streptomycin solution containing 10,000 U/ml of penicillin and 10,000 µg/ml of streptomycin (Gibco/Thermo Fisher Scientific) and 10% fetal bovine serum (Sigma-Aldrich, F4135). Cultures were passaged every 3–4 days. For voltage-clamp experiments, cells were plated on round glass coverslips with a diameter of 25 mm (VWR) in 6-well plates (Corning). At approximately 40% confluency, cells were transfected with 0.5 µg of plasmid DNA per well using Effectene Transfection Reagent (Qiagen) according to the instructions of the manufacturer. Plasmids contained the respective ion channel α subunit isoform linked by a *T2A* sequence to the expression of enhanced green fluorescent protein (EGFP) in an *Ac5-STABLE2-neo* backbone (addgene #32426) (16). The expression of ion channel β subunits was linked to *mCherry*. Fluorescent cells were used in patch clamp experiments between 48 and 72 hours after transfection.

Patch clamp experiments

A cell-covered coverslip was submerged in extracellular solution containing 5 mM TES, 103 mM NaCl, 3 mM KCl, 26 mM $NaHCO_3$, 1 mM NaH_2PO_4, 1.5 mM $CaCl_2$, 4 mM $MgCl_2$, 10 mM trehalose, 10 mM glucose and 7 mM sucrose (280 mOsM, equilibrated with 5% CO_2 and 95% O_2) in a custom-built perfusion chamber. Patch clamp pipettes (10–12 MΩ) were fabricated from borosilicate glass capillaries (BF150-86-10, Sutter Instruments) using a PC-100 micropipette puller (Narishige) and filled with intracellular solution (pH 7.3) containing 10 mM HEPES, 140 mM potassium aspartate, 1 mM KCl, 4 mM MgATP, 0.5 mM Na_3GTP and 1 mM EGTA (265 mOsM).

Pipettes were targeted at fluorescent cells using an InVivo SliceScope epifluorescence microscope (Scientifica) equipped with a 60×/1.0 NA water-immersion objective (LUMPLFLN60XW, Olympus) and an LQHXP120 light source (Leistungselektronik Jena).

Signals were recorded in the whole-cell voltage clamp configuration at 22–23˚C on a MultiClamp 700B amplifier, lowpass-filtered and sampled at 10–50 kHz using a Digidata 1550B digitizer controlled through pCLAMP 11 software (all Molecular Devices). Data were corrected for the liquid junction potential and analysed in Python v.3.13. During recordings, series resistances were monitored but not compensated and allowed to rise by at most 20% over the course of a recording. In between voltage pulses, cells were held at a membrane potential of –70 mV. Capacitive transients and linear leak currents were subtracted using a P/4 protocol.

Steady-state activation parameters were determined by applying depolarizing 500-ms voltage pulses from a holding potential of –110 mV; test voltages covered the range to +50 mV in steps of 10 mV. For fast, inactivating sodium channels, the duration of the depolarizing voltage steps was reduced to 50 ms. Conductance values (g) were calculated by dividing the measured peak currents by the difference between the test potential and equilibrium potential of the respective ion $g = I/(V–V_{Eq})$, normalized to the maximum conductance (g_{max}) of the respective cell and plotted against the test potential (V). Activation curves were fit to the function $g/g_{max} = 1/(1+e^{(V50–V)\ k})$ using the optimize.minimize function of the SciPy v.1.15 module to determine the half-maximal activation voltage ($V50$) and slope factor (k).

Steady-state inactivation parameters were obtained with the help of a two-pulse protocol, in which a pre-pulse of 500 ms (–120 to –30 mV in 5 mV increments) was followed by a 200-ms test pulse to +50 mV. Sodium currents were recoded using a pre-pulse of 100 ms and a test pulse to 0 mV of 50 ms. Peak currents (I) were normalized to the maximum current amplitude (I_{max}) of the respective cell and plotted against the pre-pulse potential (V). Curves were fit to the function $I/I_{max} = 1/(1+e^{(V–V50)\ k})$ to determine the half-maximal inactivation voltage ($V50$) and slope factor (k). To estimate the inactivation time constant, exponential functions were fit to the decaying phase of currents elicited by depolarizing voltage pulses of 500 ms, or 50 ms in case of Para. For Sh, double exponential functions were used. For the functional form of time constants over voltage, we used a bell-shaped curve whose parameters were fitted to the individual time constants.

Figures

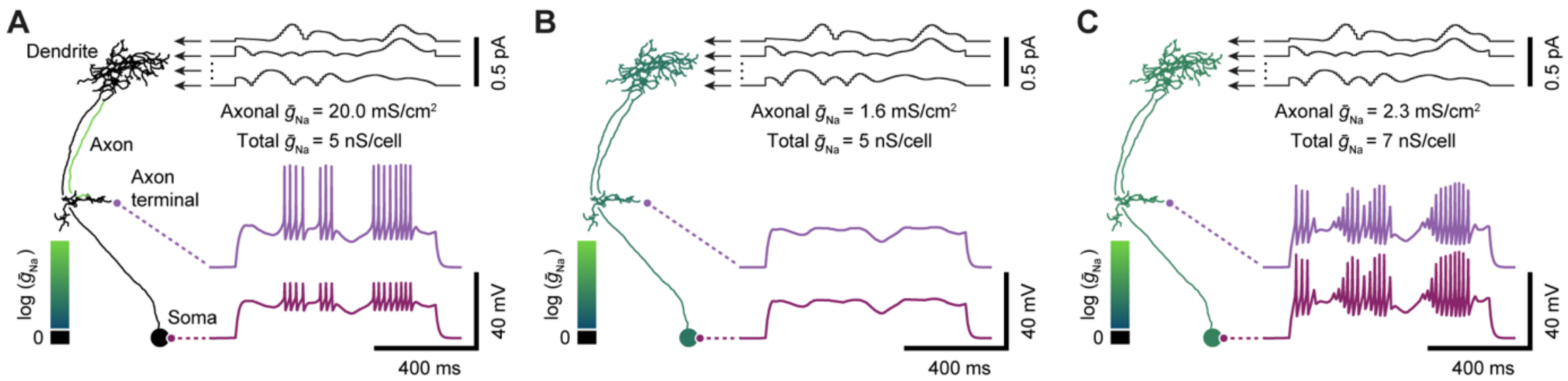

Fig. S1. Distinct sodium channel localizations give rise to distinct spike patterns in a T4 neuron model. (*A*) Hodgkin–Huxley-type compartmental model of a T4 neuron with sodium channels confined to the axon. Fluctuating depolarizing currents injected into 32 different dendritic locations (top) evoked distinct spike patterns that propagated to the axon terminal (purple) and the soma (magenta). (*B*) Same as (*A*) for a homogenous distribution of channels. Dendritic current injections did not elicit spikes despite identical total $\bar{g}_{Na}$. (*C*) The conductance of uniformly distributed sodium channels was tuned but failed to reproduce the activity pattern of (*A*). Neurite color corresponds to local sodium conductance density ($\bar{g}_{Na}$).

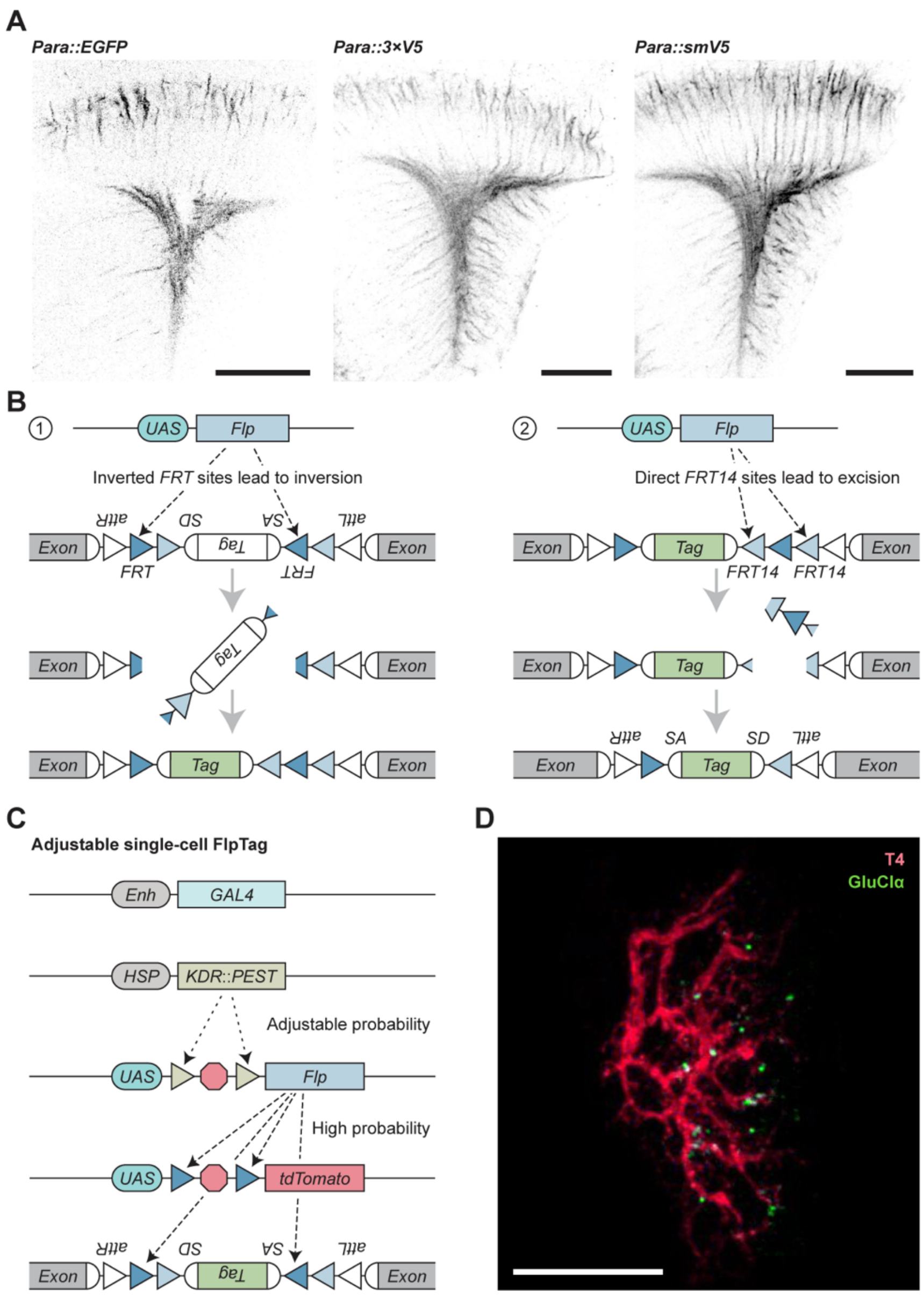

Fig. S2. Endogenous tags. (*A*) Grayscale confocal cross sections of the optic lobes of flies whose endogenous *para* locus was tagged with enhanced green fluorescent protein (left), three V5 epitopes (middle) or a protein scaffold bearing twelve V5 epitopes (right). Scale bars, 20 μm. (*B*) Irreversible activation of the FlpTag relies on a two-step process consisting of cassette inversion (left) followed by the excision (right) of one FRT site in the presence of the recombinase flippase (Flp). (*C*) Stochastic labeling of proteins in single cells of a genetically designated population was achieved by making Flp expression contingent on the excision of a stop codon by the short-lived KD recombinase (*KDR::PEST*), whose own expression was under the control of a heat shock promoter (*HSP*). (*D*) Dendrite of one individual T4 neuron (red) with native GluClα receptors visible in green. Scale bar, 10 μm.

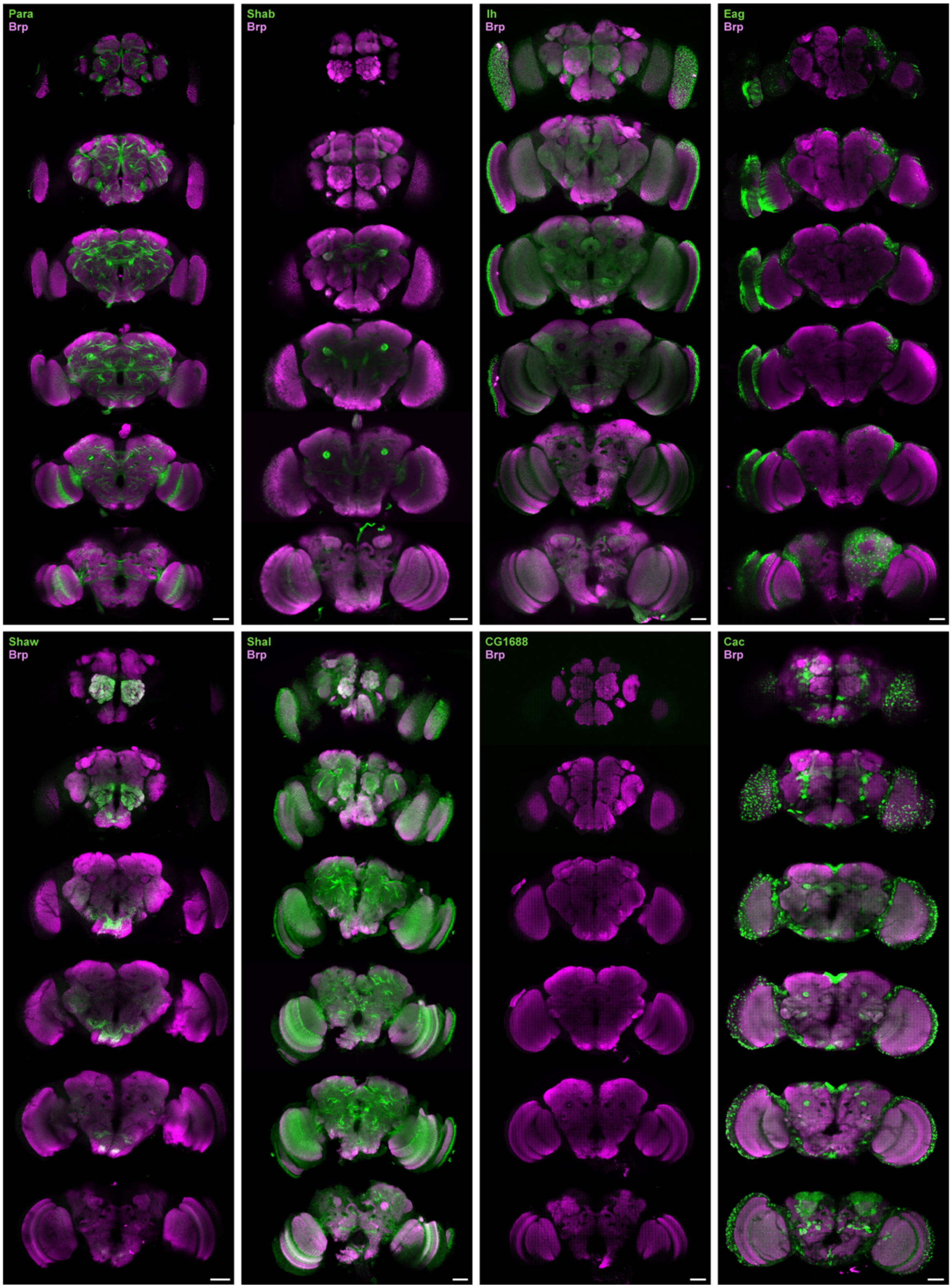

Fig. S3: Distribution of voltage-gated ion channels in the *Drosophila* brain. Endogenous ion channels marked by constitutive tags (green) and presynaptic Bruchpilot (Brp, magenta) in frontal confocal cross sections of *Drosophila* brains. The localization of Cacophony (Cac) was assessed using an overexpression construct (18). Scale bars, 50 µm.

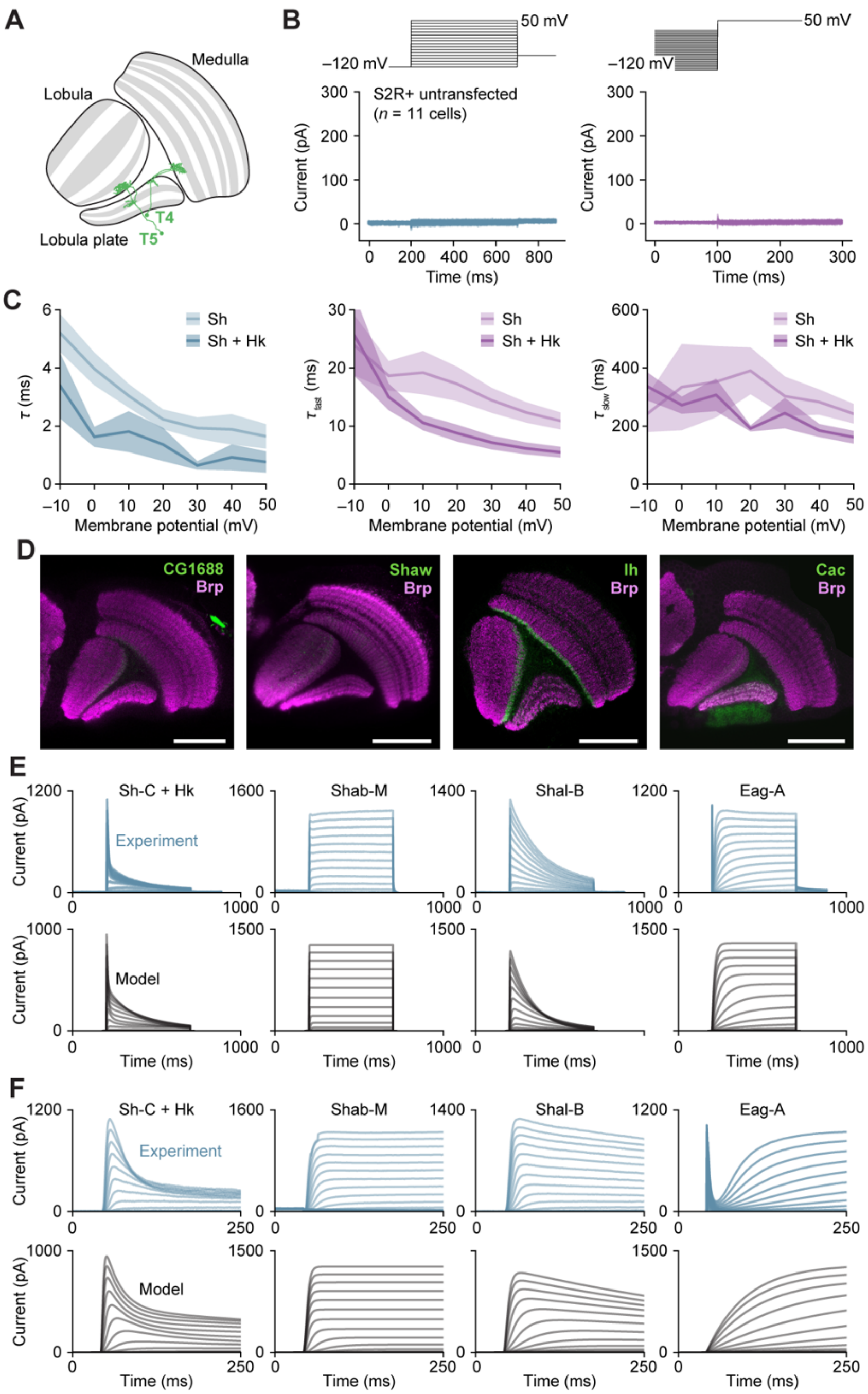

Fig. S4. Ion channels of T4 and T5 neurons. (*A*) Schematic horizontal section of the optic lobe with a one exemplary T4 and T5 neuron, each in green. (*B*) Average whole-cell currents (bottom) measured in response to voltage pulses (top) in native S2R+ cells. (*C*) Voltage dependence of activation time constants (left) and fast (middle) and slow (right) inactivation time constants of whole-cell currents measured in response to square-wave voltage pulses in S2R+ cells transfected with Sh or co-transfected with Sh and Hk. (*D*) Confocal cross sections of the optic lobe with endogenously tagged channels (green) and Bruchpilot (Brp, magenta) to mark synapses. Scale bars, 50 µm. (*E*) The gating parameters of potassium channel models were derived by fitting simulated (black) to measured (blue) currents. (*F*) Same as (*D*) but with an expanded time axis for better comparison.

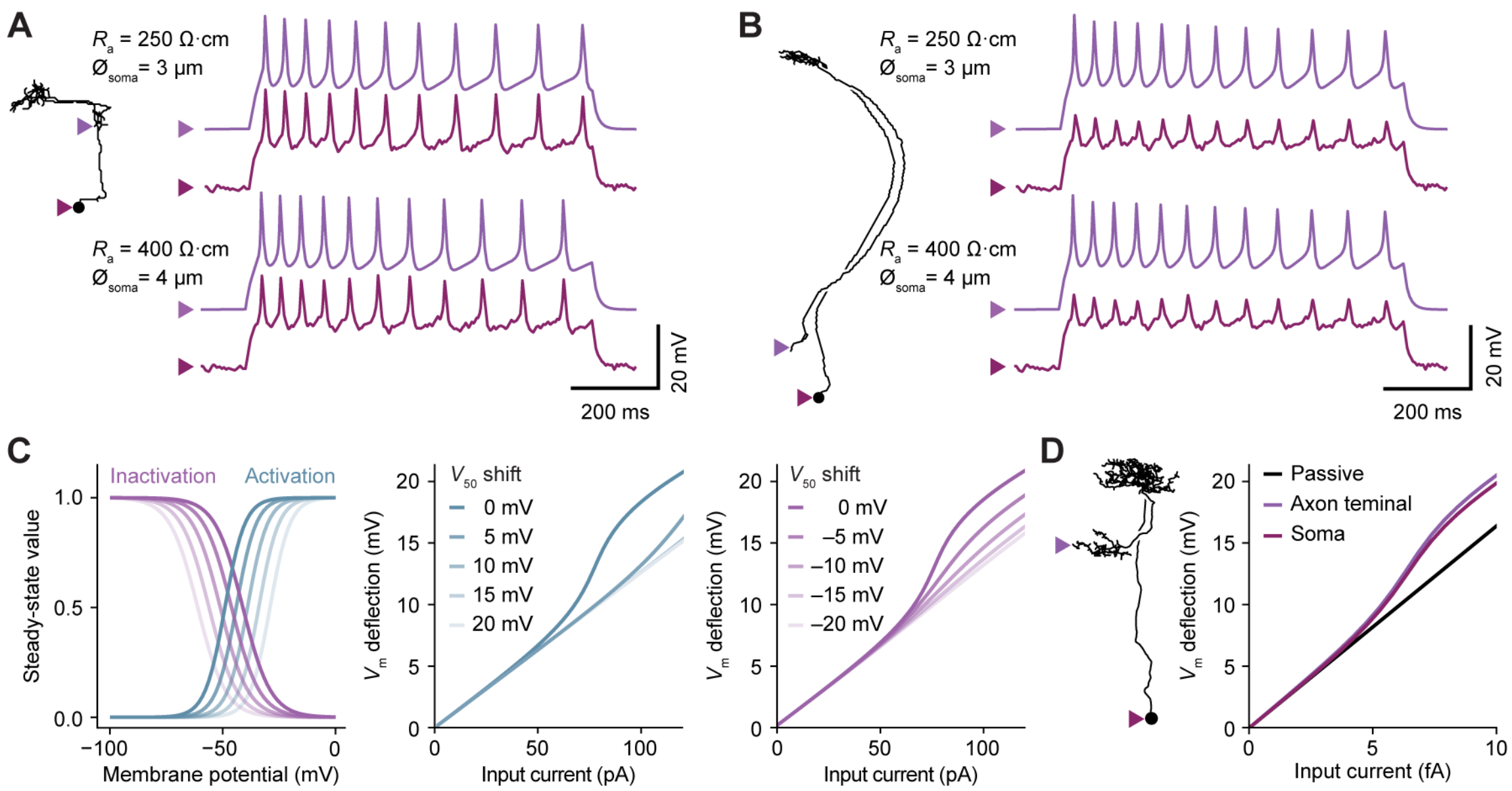

Fig. S5. Effects of morphology and Para gating on spike propagation and response nonlinearity. (*A*) Short-neurite T4 neuron models with 'spiking' axonal sodium channels and different axial resistivity (R_a) and soma diameter ($\varnothing_{soma}$). Input-evoked action potentials were detectable in the axon terminal (purple) and the soma (magenta). Triangles indicate recording sites. (*B*) Same as (*A*) but for a T4 neuron with a long neurite. (*C*) Artificial shifts in half-maximal steady-state activation and inactivation curves (left) and their influence on the simulated membrane potential (V_m) deflection as a function of input current (middle and right). (*D*) Simulated V_m deflection as a function of input current recorded in the axon (purple) and in the soma (magenta) of a compartmental T5 neuron model. V_m deflections of a passive neuron are shown for reference (black).

Tables

Table S1. Insertion sites of endogenous tags.

Gene	Chromosome	Insertion site	Isoforms captured	Fraction of isoforms captured
Sh	X	MI10885	*A, B, C, D, E, F, H, I, J, K, L, M, Q, T, U, V*	16/17
Shal	3	MI00446	*A, B, C*	3/3
Shab	3	MI00848	*A, B, C, H, I, J, L, M, N*	9/10
Shaw	2	MI01735	*B, C, E*	3/5
eag	X	CR01421	*A, B, C, D, E, F, G*	7/7
CG1688	2	CR01570	*A*	1/1
Ih	2	MI12136	*B, C, E, F, G, H, I, J, K, L, M*	11/11
para	X	MI08578	*A, AA, AB, AC, AD, AE, AF, AG, AH, AI, AJ, AK, AL, AM, AN, AO, AP, AQ, AR, AS, AT, AU, AV, AW, AX, AY, AZ, B, BA, BB, BC, BD, BE, BF, BG, BH, C, D, E, F, G, H, I, J, K, L, M, N, O, P, Q, R, S, T, U, V, W, X, Y, Z*	60/60

Table S2. Resources

Fly strain/plasmid	Research resource identifier (RRID)
y[1]w[*]; Mi{FlpTag.1}Shab[MI00848-FT.1-X]	BDSC_606263
y[1]w[*]; Mi{FlpTag.1}Shab[MI00848-FT.1]	BDSC_606264
y[1]w[*]; Mi{FlpTag.1}Shal[MI00446-FT.1-X]	BDSC_606265
y[1]w[*]; Mi{FlpTag.1}Shal[MI00446-FT.1]	BDSC_606266
y[1]w[*]; TI{FlpTag.1}CG1688[CR01570-FT.1-X]/CyO	BDSC_606267
y[1]w[*]; TI{FlpTag.1}CG1688[CR01570-FT.1]/CyO	BDSC_606268
y[1]w[*]; Mi{FlpTag.2}Sh[MI10885-FT.1-X]	BDSC_606269
y[1]w[*]; Mi{FlpTag.2}Sh[MI10885-FT.1]	BDSC_606270
y[1]w[*]; TI{FlpTag.1}eag[CR01421-FT.1-X]	BDSC_606271
y[1]w[*]; TI{FlpTag.1}eag[CR01421-FT.1]	BDSC_606272
y[1] w[*]; Mi{FlpTag.smV5.2}Ih[MI12136-FT.2-X]/CyO	BDSC_606273
y[1] w[*]; Mi{FlpTag.smV5.2}Ih[MI12136-FT.2]/CyO	BDSC_606274
y[1]w[*]; Mi{FlpTag.smV5.2}Sh[MI10885-FT.1-X]	BDSC_606275
y[1]w[*]; Mi{FlpTag.smV5.2}Sh[MI10885-FT.1]	BDSC_606276
y[1]w[*]; Mi{FlpTag.smV5.1}Shab[MI00848-FT.1-X]/TM3, Sb[1] Ser[1]	BDSC_606277
y[1]w[*]; Mi{FlpTag.smV5.1}Shab[MI00848-FT.1]/TM3, Sb[1] Ser[1]	BDSC_606278
y[1]w[*]; TI{FlpTag.smV5.1}eag[CR01421-FT.1-X]	BDSC_606279
y[1]w[*]; TI{FlpTag.smV5.1}eag[CR01421-FT.1]	BDSC_606280
y[1]w[*]; TI{FlpTag.smV5.1}CG1688[CR01570-FT.1-X]	BDSC_606281
y[1]w[*]; TI{FlpTag.smV5.1}CG1688[CR01570-FT.1]	BDSC_606282
y[1]w[*]; Mi{FlpTag.smV5.1}Shal[MI00446-FT.1-X]/TM3, Sb[1] Ser[1]	BDSC_606283
y[1]w[*]; Mi{FlpTag.smV5.1}Shal[MI00446-FT.1]/TM3, Sb[1] Ser[1]	BDSC_606284
y[1]w[*]; Mi{FlpTag.smV5.1}Shaw[MI01735-FT.1-X]/CyO	BDSC_606285
y[1]w[*] Mi{FlpTag.smV5.1}Shaw[MI01735-FT.1]/CyO	BDSC_606286
y[1] w[*]; Mi{FlpTag.smV5.0}para[MI08578-FT.0-X]	BDSC_606702
y[1] w[*]; Mi{FlpTag.smV5.0}para[MI08578-FT.0]	BDSC_606703
w[1118]; P{y[+t7.7] w[+mC]=10XUAS(D6rox.stop)FLP}su(Hw)attP5; P{y[+t7.7] w[+mC]=3XUAS-Dre.PEST}su(Hw)attP1/TM6B, Tb[1]	BDSC_606704
w[1118]; P{y[+t7.7] w[+mC]=10XUAS(FRT.stop)CD4::tdTomato.V}su(Hw)attP5/CyO	BDSC_606708
pUC57-FlpTag-smV5-ph0	Addgene_232876
pUC57-FlpTag-smV5-ph1	Addgene_232877
pUC57-FlpTag-smV5-ph2	Addgene_232878
pUC57-FlpTag-3xV5-ph0	Addgene_232873
pUC57-FlpTag-3xV5-ph1	Addgene_232874
pUC57-FlpTag-3xV5-ph2	Addgene_232875

SI References

1. M. Deistler, *et al.*, Jaxley: differentiable simulation enables large-scale training of detailed biophysical models of neural dynamics. *Nat. Methods* **22***, 2649–2657* (2025).
2. K. Shinomiya, *et al.*, Comparisons between the ON- and OFF-edge motion pathways in the *Drosophila* brain. *eLife* **8**, e40025 (2019).
3. M. L. Hines, N. T. Carnevale, Neuron: a tool for neuroscientists. *Neuroscientist* **7**, 123–135 (2001).
4. A. Nern, *et al.*, Connectome-driven neural inventory of a complete visual system. *Nature* **641**, 1225–1237 (2025).
5. M. Meier, A. Borst, Extreme compartmentalization in a *Drosophila* amacrine cell. *Curr. Biol.* **29**, 1545-1550.e2 (2019).
6. A. Borst, J. Haag, The intrinsic electrophysiological characteristics of fly lobula plate tangential cells: I. Passive membrane properties. *J. Comput. Neurosci.* **3**, 313–336 (1996).
7. N. W. Gouwens, R. I. Wilson, signal propagation in *Drosophila* central neurons. *J. Neurosci.* **29**, 6239–6249 (2009).
8. L. N. Groschner, J. G. Malis, B. Zuidinga, A. Borst, A biophysical account of multiplication by a single neuron. *Nature* **603**, 119–123 (2022).
9. M. Pospischil, *et al.*, Minimal Hodgkin–Huxley type models for different classes of cortical and thalamic neurons. *Biol. Cybern.* **99**, 427–441 (2008).
10. J. Haag, A. Arenz, E. Serbe, F. Gabbiani, A. Borst, Complementary mechanisms create direction selectivity in the fly. *eLife* **5**, e17421 (2016).
11. A. Borst, A biophysical mechanism for preferred direction enhancement in fly motion vision. *PLoS Comput. Biol.* **14**, e1006240 (2018).
12. J. R. Bateman, A. M. Lee, C. Wu, Site-specific transformation of *Drosophila* via ϕC31 integrase-mediated cassette exchange. *Genetics* **173**, 769–777 (2006).
13. K. J. T. Venken, *et al.*, MiMIC: a highly versatile transposon insertion resource for engineering *Drosophila melanogaster* genes. *Nat. Methods* **8**, 737–743 (2011).
14. O. Kanca, *et al.*, An expanded toolkit for *Drosophila* gene tagging using synthesized homology donor constructs for CRISPR-mediated homologous recombination. *eLife* **11**, e76077 (2022).
15. S. Fendl, R. M. Vieira, A. Borst, Conditional protein tagging methods reveal highly specific subcellular distribution of ion channels in motion-sensing neurons. *eLife* **9**, e62953 (2020).
16. M. González, *et al.*, Generation of stable *Drosophila* cell lines using multicistronic vectors. *Sci Rep* **1**, 75 (2011).
17. F. P. Davis, *et al.*, A genetic, genomic, and computational resource for exploring neural circuit function. *eLife* **9**, e50901 (2020).
18. F. Kawasaki, B. Zou, X. Xu, R. W. Ordway, Active zone localization of presynaptic calcium channels encoded by the *cacophony* locus of *Drosophila*. *J. Neurosci.* **24**, 282–285 (2004).
